# Mannose-functionalization of reconstituted high-density lipoprotein nanoparticles improves payload delivery and enhances M2-to-M1 phenotype reprogramming of RAW 264.7 macrophages polarized by B16-F10 melanoma cells

**DOI:** 10.3389/fddev.2023.1281066

**Published:** 2023-10-24

**Authors:** Akpedje S. Dossou, Morgan E. Mantsch, Nirupama Sabnis, Rance E. Berg, Rafal Fudala, Andras G. Lacko

**Affiliations:** ^1^ Lipoprotein Drug Delivery Lab, Department of Microbiology, Immunology and Genetics, UNT Health Science Center, Fort Worth, TX, United States; ^2^ University of Texas at Austin, College of Natural Sciences, Austin, TX, United States; ^3^ Department of Microbiology, Immunology and Genetics, UNT Health Science Center, Fort Worth, TX, United States

**Keywords:** mannose, rHDL, DMXAA, paclitaxel, CD206, SR-B1, B16-F10, RAW 264.7

## Abstract

The targeting and conversion of the immunosuppressive (M2) tumor-associated macrophages (TAMs) to an immunostimulatory (M1) phenotype can induce tumor regression in advanced melanoma. We have previously characterized and reported the ability of reconstituted high-density lipoprotein nanoparticles (rHDL NPs) functionalized with DSPE-PEG-mannose (DPM) to deliver payload to macrophages. Herein, we investigate the modulation of macrophage phenotype and payload delivery mechanisms of the rHDL-DPM NPs in RAW 264.7 murine macrophages exposed to the conditioned medium (CM) from murine B16-F10 melanoma cells. The rHDL-DPM NPs loaded with the Stimulator of Interferon genes agonist, DMXAA, reduced protein levels of M2 markers. Through the mannose moiety, the rHDL-DPM-DMXAA NPs enhanced the production of interferon β and CXCL10 compared to the free DMXAA in the B16-F10 CM-educated RAW 264.7 macrophages. Compared to their non-mannosylated counterpart, the rHDL-DPM NPs delivered their payload more efficiently to the B16-F10 CM-educated RAW 264.7 macrophages. Mechanistically, both the scavenger receptor type B class 1 (SR-B1) and the mannose receptor (CD206) facilitated payload delivery to the macrophages via endocytic and non-endocytic mechanisms. Finally, the CM from rHDL-DPM-DMXAA NPs -treated macrophages enhanced paclitaxel (paclitaxel)-mediated cytotoxicity in B16-F10 cells. Together, these *in vitro* findings demonstrate the potential of the mannose-functionalized rHDL NPs in improving the targeting of M2-like TAMs and treatment outcomes when combined with immunotherapy or PTX in B16-F10 melanoma *in vivo* models.

## 1 Introduction

Although melanoma is the least common type of skin cancer, it has a rapid growth rate, a propensity to metastasize, and accounts for most skin cancer-related deaths. If melanoma is left to progress to metastasis, the chances of survival become are substantially reduced (about 30% as opposed to 99% for melanoma *in situ*) (American Cancer Society, 2023a). Skin excision is effectively used to treat early stage localized melanoma (American Cancer Society, 2022b). Melanoma treatment, however, becomes more challenging once the cancer has gained metastatic features (Sundararajan et al., 2022). The advent, refinement and clinical application of immunotherapies and targeted therapies in the last two decades have improved the 5-year overall survival of advanced melanoma that were otherwise dismal with standard chemotherapeutic treatments (Yu et al., 2019; Frampton and Sivakumar, 2022). The improved response rate to treatment via immune checkpoint inhibitors over chemotherapeutic drugs highlights the importance of the immune system in halting tumor progression (CiRen et al., 2016). Nevertheless, over the course of therapy, the therapeutic response rate can drop in advanced melanoma patients and relapse can occur (Sambi et al., 2019; Dobosz et al., 2022).

While the reduced response to therapy is multifactorial, the immunosuppression induced by the abundant M2-like TAMs in the melanoma tumor microenvironment (TME) plays a significant role in impeding the efficacy of immunotherapies and in aiding the progression of the disease (Pieniazek et al., 2018). The M2-like TAMs can turn tumors cold with paucity in infiltration of professional antigen-presenting cells and cytotoxic immune cells, including natural killer cells and CD8^+^ T cells (Bonaventura et al., 2019; Ceci et al., 2020; Dobosz et al., 2022). Several studies in murine models of melanoma have demonstrated that the re-education of the M2-TAMs to an M1-like (immunostimulatory) phenotype can improve the efficacy of immunotherapy and leads to tumor regression (Ceci et al., 2020). Agonism of the STimulator of INterferon Genes (STING) in macrophages can promote an M1 phenotype (Wang et al., 2022). This approach has shown benefit in eliciting anticancer adaptive immune response and in synergizing with immunotherapy or chemotherapy (Corrales et al., 2015; Chipurupalli et al., 2020; Chelvanambi et al., 2021). Due to the whole body-distribution of macrophages, there is a need to specifically deliver M2-to-M1 reprogramming agents, such as STING agonists, to macrophages at the tumor site to avoid adverse effects on the immune system. Additionally, M2-to-M1 reprogramming agents administered intratumorally may not impact macrophages at metastatic sites (Marabelle et al., 2018; Meric-Bernstam et al., 2022). The functionalization of payload-carrying nanoparticles (NPs) with moieties that recognize highly expressed receptors on TAMs-including the mannose receptor CD206-can improve specific TAM targeting (He et al., 2021).

Reconstituted high-density lipoprotein (rHDL) NPs have been utilized successfully to transport hydrophobic drugs or imaging agents (Raut et al., 2018; Mei et al., 2021). Their assembly rests upon the well-established high affinity of the apolipoprotein A-I (ApoA-I) for lipids (Koyama et al., 2009; Gorshkova et al., 2014). They are biocompatible, generally well-tolerated in patients and can easily penetrate and accumulate in the tumor mass (Tardif et al., 2007; Simonsen, 2016; Ma et al., 2018; Raut et al., 2018). With respect to their ability to deliver payload to TAMs, radiolabeled rHDL NPs targeted macrophages more effectively than other cells in the TME (Perez-Medina et al., 2015; MacCuaig and McNally, 2020). Moreover, drug-loaded synthetic HDL NPs showed a high specificity for M2-TAMs at the tumor site (Wang et al., 2021; Zheng et al., 2022; Xiong et al., 2023), suggesting that HDL-inspired NPs can help achieve precise targeting of TAMs. We have previously optimized and characterized mannose-functionalized rHDL NPs where rHDL NPs were modified with 1,2-distearoyl-sn-glycero-3-phosphoethanolamine-N-(polyethyleneglycol, 2K)-Mannose (DSPE-PEG-Mannose or DPM). The rHDL-DPM NPs not only were able to deliver 5,6-dimethylxanthenone-4-acetic acid (DMXAA), a STING agonist, to macrophages, but they were also able to modulate macrophage phenotype (Dossou et al., 2023).

In the present study, we evaluated and investigated macrophage phenotype modulation and payload delivery by the rHDL-DPM-DMXAA NPs using RAW 264.7 murine macrophages exposed to the conditioned medium (CM) from B16-F10 murine melanoma cells. Since HDL-type NPs can target macrophages and melanoma-associated macrophages express CD206 (Tham et al., 2014; Qian et al., 2017), we hypothesized that the rHDL-DPM-DMXAA NPs would induce an M1 phenotype in B16-F10 CM-educated RAW 264.7 macrophages, and that the rHDL-DPM NPs would deliver their payload via the HDL receptor, scavenger receptor class B type 1 (SR-B1). Our *in vitro* results indicate that the rHDL-DPM NPs deliver their payload majorly via both SR-B1 and CD206 and can enhance the M2-to M1 reprogramming in macrophages. In addition, the rHDL-DPM NPs indirectly improve the sensitivity of B16-F10 cells to the chemotherapeutic drug paclitaxel (PTX). The findings of this study confirmed our hypothesis and underscore the potential of the rHDL-DPM NPs to improve TAM targeting and to enhance treatment outcome *in vivo* mouse models of B16-F10 melanoma, at least when combined with immunotherapy or with PTX.

## 2 Materials and methods

### 2.1 Materials

The B16-F10 melanoma cells (Cat# CRL-6475) and RAW 264.7 mouse macrophages (TIB-71) were acquired from the American Type Culture Collection (ATCC, Manassas, VA, United States). The Invitrogen recombinant mouse interleukin-4 (rm IL-4, Cat# PMC0045), Invitrogen rm interferon-gamma (rm IFNγ, Cat# BMS326), Invitrogen lipopolysaccharide (LPS, Cat# 00-4976-93), anti-arginase 1 (Arg1) rabbit monoclonal antibody (Cat# 702730), rabbit IgG isotype control (Cat# 10500C), Hoechst nuclear stain solution (Cat# 62249), phosphate-buffered saline, pH 7.4 (PBS, Cat# 10010), Alexa Fluor 488 carboxylic acid, succinimidyl ester (AF488 NHS, Cat# A20100), mouse IP-10 (also called C-X-C motif chemokine ligand 10, CXCL10) ELISA kit (Cat# BMS6018), western blot and cell culture supplies were purchased from Thermo Fisher Life Technologies Corporation (Carlsbad, CA, United States). The anti-CD206 rabbit monoclonal antibody (Cat# NBP2-66956), the mouse IFNβ ELISA kit (Cat# MIFNB0) and the mouse tumor necrosis factor alpha (TNFα) ELISA kit (Cat# MTA00B) were acquired from R&D Systems (Minneapolis, MN, United States). The secondary antibodies (all HRP-linked) goat anti-mouse IgG (Cat# 7076S), goat anti-rabbit IgG (Cat# 7074S) were obtained from Cell Signaling Technology (Danvers, MA, United States) and the donkey anti-rabbit IgG (Cat# 711-035-152) was purchased from Jackson ImmunoResearch Laboratory, Inc. (West Grove, PA, United States). PTX (Cat# HY-B0015) and DMXAA (Cat# HY-10964) were obtained from MedChemExpress (Monmouth Junction, NJ, United States). The Poly-D-lysine-coated glass-bottom 35 mm dishes (Cat# P35GC-1.5-10-C) were obtained from the MatTek Corporation (Ashland, MA, United States). Inorganic and organic chemicals (unless otherwise stated) as well as mannan (product #M7504), D- + -mannose (product #M8574), D- + -glucose (product #G7021), Cytochalasin D (CytD product #C8273), block lipid transport-1 (BLT-1, product # 373210), mouse monoclonal anti-β-actin (product # A5441), Nile Red (NR, product # 72485), free cholesterol (FC, product #C8667), egg yolk L-α-phosphatidyl choline (EYPC, product # 61755) were purchased from Sigma-Aldrich Corporation (St Louis, MO, United States). Cerenis Therapeutics-now Abionyx Pharma- (Balma, France) supplied the ApoA-I (batch #2451PF41) which was produced endotoxin -free in Chinese hamster ovarian cells. The DSPE-PEG(2K)-mannose or DPM (Cat# LP096282, Cat ID: 12,169) was purchased from Biopharma PEG Scientific Inc. (Watertown, MA, United States). The DSPE-PEG (Cat# MPL0301) was purchased from Advanced BioChemicals, LLC (Lawrenceville, GA, United States). The cytotoxicity CCK8 Kit were obtained from Dojindo Molecular Technologies, Tubaru, Japan.

### 2.2 Methods

#### 2.2.1 Synthesis of mannose-functionalized rHDL NPs

The NPs were synthesized as previously described (Dossou et al., 2023). Briefly, egg yolk phosphatidylcholine (EYPC), free cholesterol (FC) and DSPE-PEG-mannose (DPM) chloroform solutions were mixed in a liquid scintillation glass vial and dried under a stream of nitrogen gas until formation of a thin film. After rehydration of the thin film with PBS, the payload (either DMXAA or Nile Red) in powder form was added to the mixture. The mixture was then vortexed and sonicated for 2 min, with 3 min rest on ice for 30 min at amplitude 80. ApoA-I in 6M guanidine hydrochloride was added dropwise to the emulsion, and the mixture constituted of EYPC, ApoA-I, FC, DPM in the molar ratio of 100:1:10:2 with or without payload was left to incubate overnight with rotatory shaking at 4°C in the dark. Then, the preparation was transferred to a 50 KDa dialysis bag and dialyzed against PBS for 6 h at 4°C in the dark. After dialysis and centrifugation at 12,000 rpm for 30 min at 4°C, the preparation was filter-sterilized through a 0.2 µM syringe filter and keep at 4°C in the dark. Particles assembled without DPM were made and are referred to as rHDL NPs. While the formulations with DMXAA and the Nile Red (NR) dye are referred to respectively as rHDL-DPM-DMXAA NPs and rHDL-DPM-NR NPs, the empty formulations with DPM are referred to as rHDL-DPM NPs. The same preparation workflow was utilized for all variations of the particles (DSPE-PEG instead of DPM, no ApoA-I, or no DPM or just DPM micelles). Fresh preparations of particles were utilized for all studies, and characterized new preparations were made for each replicate of all the studies conducted.

#### 2.2.2 Synthesis of Alexa Fluor 488-ApoA-I labeled-NPs

To label ApoA-I, 1 mL of 0.1M sodium bicarbonate was added to 1 mL of 17.7 mg/mL ApoA-I. Then, 5 mg of AF488 NHS, ester was dissolved in 0.5 mL DMSO and 100 µL of the resulting dye solution was added to the ApoA-I solution. The reaction was conducted as per the protein labeling kit manufacturer instructions. At the end of the reaction, the mixture was applied to a PD10 column, and 1 mL-fractions were collected. A bicinchoninic acid (BCA assay) was used to detect the labeled protein in the eluted fractions and to separate the labeled protein from the free AF488 dye. Absorbance measurements for the degree of labeling were carried out as recommended in the manual using a spectrophotometer. The AF488-labeled ApoA-I was added to the DPM-containing lipid mix as described in the above section for the unlabeled ApoA-I.

#### 2.2.3 Characterization of the NPs

The particle diameter size, polydispersity index (PDI), and zeta potential were acquired using the Malvern light scattering system Zetasizer Ultra and the ZS Xplorer software (Malvern Panalytical Ltd., Malvern, United Kingdom). The DMXAA was quantified via absorbance measurement at 350 nm. The retention of DPM or DSPE-PEG in the formulations was indirectly assessed to ensure at least 90% of DPM retention, using a barium chloride/iodide assay to detect PEG to as previously described (Chung et al., 2000; Dossou et al., 2023). Using the weight (W) of payload, the entrapment efficiency (EE) and drug loading (DL) pertaining to DMXAA were calculated as follows:

$$EE={Mass}_{DMXAA\;recovered\;in\;formulation}\times 100\;\%\;/\;{Mass}_{DMXAA\;initially\;added}$$

$$DL={Mass}_{DMXAA\;recovered\;in\;formulation}\times 100\;\%\;/{Mass}_{total\;formulation}$$

#### 2.2.4 *In vitro* studies

##### 2.2.4.1 Cell culture conditions

The B16-F10 melanoma cells and RAW 264.7 macrophages were cultured throughout the treatments at 37°C in 5% CO2 in a humidified incubator. Both the B16-F10 and the RAW 264.7 macrophages were maintained in complete DMEM (DMEM media supplemented with 10% FBS and 1% Pen Strep). For the experiments, B16-F10 cells from passage 5 to passage 28 and RAW 264.7 macrophages from passage 3 to passage 12 (after they were obtained from vendors) were used. The cell lines were routinely tested for *mycoplasma* contamination using the MycoFluor™ *Mycoplasma* Detection Kit (Cat#M7006, Thermo Fisher Life Technologies Corporation) and found to be negative for *mycoplasma* throughout the study.

##### 2.2.4.2 Collection of conditioned media

For experiments with CM from cancer cells, 2 × 10^6^ B16-F10 cells were seeded in T-75 flasks and maintained in complete DMEM (cDMEM) till 80%–90% confluency. Then, the culture media was collected and centrifuged at 1,500 rpm for 5°min. The supernatant was filtered through a sterile 0.45 µM-pore size filter and stored at −80°C until use. For experiments assessing the effect of the CM from the NPs-treated RAW 264.7 macrophages on the sensitivity of B16-F10 cells to PTX, the RAW 264.7 macrophages were washed twice with PBS to remove the pre-treatments with the different formulations. Then, the cells were left to incubate in cDMEM for 12 h. After incubation, the same CM collection protocol was used.

##### 2.2.4.3 RAW 264.7 polarization and treatment with NPs

The polarization of RAW 264.7 macrophages and CM treatments were performed as previously described (Liu et al., 2013; Hwang et al., 2020; Chong et al., 2022; Dossou et al., 2023). Briefly, 2 × 10^6^ RAW 264.7 macrophages were seeded in a 60 mm dish and were allowed to attach overnight. The RAW 264.7 macrophages in cDMEM were stimulated for 24 h with either 50 ng/mL LPS+20 ng/mL IFNγ to generate the M1 phenotype or with 20 ng/mL IL-4 to generate the M2 phenotype. To generate B16-F10 CM-educated macrophages, the seeded RAW 264.7 macrophages were maintained in cDMEM supplemented with 20% of B16-F10 CM for up to 48 h. For treatment with the NPs, the CM-educated RAW 264.7 macrophages were treated for 24 h with an equivalent amount of 20°ug/mL DMXAA for free DMXAA (DMXAA dissolved in 7.5% sodium bicarbonate), rHDL-DPM-DMXAA NPs, rHDL-DSPE-PEG-DMXAA NPs, and associated controls such as vehicle (7.5% sodium bicarbonate), rHDL NPs, rHDL-DPM NPs, rHDL-DSPE-PEG NPs, ApoA-I, EYPC-FC micelles and DPM micelles. Untreated cells were kept as control. All formulations used for cellular treatments were filter-sterilized through a sterile 0.2 µm-syringe filter. The rHDL-DMXAA NPs were not included in the study due to low DMXAA retention (Dossou et al., 2023). All the treatments were administered in CM-supplemented cDMEM. For studies involving BLT-1, the cells were pre-incubated for 1 h with 1 µM BLT-1 before addition of treatments and during exposure to treatments. After the 24 h incubation with the treatments, the supernatants were collected and subjected to ELISA while the cells were collected to quantify protein levels via western blot.

##### 2.2.4.4 B16-F10 cell treatments

To investigate the effect of the NPs on cytokine production by B16-F10 cells, 2 × 10^6^ B16-F10 cells were seeded in a 60 mm dish and allowed to attach overnight. Then, the B16-F10 cells were rinsed with PBS and treated with vehicle, Free DMXAA, rHDL-DPM NPs or rHDL-DPM-DMXAA NPs for 24 h as described for the RAW 264.7 macrophages above. To investigate the effect of the CM from the treated RAW 264.7 macrophages on the proliferation and viability of B16-F10 cells, 1 × 10^5^ B16-F10 cells were seeded in 12-well plates and allowed to attach overnight. After incubation, the cells were rinsed with PBS, and incubated in cDMEM supplemented with 50% CM from pre-treated RAW 264.7 macrophages (collected as mentioned above). The B16-F10 cells were then left to incubate for 24 h after which the cells were collected for cell counting and viability. For studies involving PTX, two concentrations, 1 μg/mL (PTX1) and 5 μg/mL (PTX5), were used to treat B16-F10 cells (Sun et al., 2021). First, 5 × 10^3^ B16-F10 cells were seeded in a 96-well plate. After attachment, they were incubated for 24 h in cDMEM supplemented with PTX1 or PTX5 and the relevant CM from the treated macrophages.

##### 2.2.4.5 Cellular payload uptake studies

To investigate the contribution of cellular receptors and nanoparticle components in the uptake of payload from the NPs, the cells were treated with NR-containing NPs as previously described (Dossou et al., 2023). 2 × 10^5^ cells (RAW 264.7 macrophages and B16-F10 cells) were seeded in a poly-D-lysine-coated 35 mm glass bottom dish and allowed to attach overnight. Then, the RAW 264.7 macrophages were treated with either B16-F10 CM or with 50 ng/mL LPS +20 ng/mL IFNγ or 20 ng/mL IL-4 or with the DMXAA-loaded particles after CM incubation as described in the earlier sections. Before the uptake studies, the cells were rinsed twice with PBS, and incubated with various formulations of NR (with an equivalent amount of 0.5 µM NR) including rHDL-DPM NR, rHDL-DSPE-PEG-NR, rHDL-NR, EYPC-FC-DPM-NR, free NR, and the labeled rHDL(ApoA-I-AF488)-DPM NPs dispersed in cDMEM. To assess the role of SR-B1, CD206 and endocytosis in cellular uptake of the nanoparticle content, the cells were pre-incubated with inhibitors at 37°C before they were exposed to different formulations of NR and during exposure to these formulations. The specific SR-B1 inhibitor, BLT-1 (Nieland et al., 2008; Yu et al., 2011), in 0.5, 1, 10, 100 µM or anti-SR-B1 antibody along with the isotype control were used to inhibit SR-B1 for 1 h. To inhibit CD206, the cells were pre-treated for 15 min with a range of mannose concentrations (0.5, 1, 2.5 and 5 mg/mL), 5 mg/mL mannan and 5 mg/mL glucose (Kato et al., 2000) or for 1 h with anti-CD206 antibody and isotype control. For studies involving the combination of BLT-1 and mannose, the mannose was added in the last 15 min of the 1-h pre-incubation with BLT-1. For endocytosis, the cells were pre-incubated for 5 h with 5 μg/mL cytochalasin D (CytD) (Francia et al., 2019). After incubation with the NR formulations, the cells were washed 3 times with PBS and either incubated with 5 µM Hoechst in PBS for 10 min followed by 3 washes of PBS and re-incubated in phenol red-free DMEM media. The visualization of the cells and the NR mean fluorescence intensity (MFI) analysis per cell were conducted using the Biotek Cytation Image reader and its cellular analysis features. The same exposure settings (intensity, integration time, camera gain) were utilized for all treated cells within an experiment to allow comparison between treatments.

##### 2.2.4.6 Cytotoxicity and cell viability studies

The cytotoxicity CCK8 Kit was utilized to evaluate cytotoxic effects of the NPs on B16-F10 CM-educated RAW 264.7 macrophages and B16-F10 cells (Dossou et al., 2023). The cytotoxicity results are presented as the percent absorbance calculated as follows: (Absorbance at 450 nm of treatments X 100)/Absorbance at 450 nm of untreated control. Treatment with the CCK8 kit reagent was performed the same way to assess cytotoxic effects on B16-F10 cells in studies involving PTX. Cytotoxicity studies pertaining to the different inhibitors were conducted using the CCK8 kit reagent for the duration of incubations in the uptake studies. For the cell viability studies or cell number determination, the treated cells were washed with PBS and detached with trypsin. After centrifugation, the sedimented cells were resuspended in 1 mL of PBS. Then, the cells were stained with 0.4% trypan blue as recommended (Strober, 2015), counted and analyzed using the trypan blue exclusion mode of the Denovix CellDrop BF cell counter (Denovix, Inc., Wilmington, DE, United States).

##### 2.2.4.7 Enzyme-linked Immunosorbent assays and immunoblotting

After the treatment period, the media from the different treatment groups of cells as described in the above sections were centrifuged (5,000 rpm, 5°min, 4°C) to remove cellular debris. The supernatants were stored at −80°C until quantification of cytokines via ELISA. The supernatants of the same samples were assayed for TNFα, IFNβ and CXCL10 following the kit’s manufacturers guidelines and including culture media in the negative controls. The values obtained were normalized to cell number determined as described above. To investigate the SR-B1, CD206, and Arg1 protein expression, the treated cells were processed as previously described (Xu et al., 2022). Briefly, the trypsinized cells were washed with ice cold PBS and lysed using the RIPA lysis buffer supplemented with 1X protease and phosphatase inhibitor cocktail with sonication at amplitude 20 for 30°s. After 30 min of incubation on ice, the lysed cells were centrifuged for 10 min at 12,000 rpm, and the supernatant was assayed for protein concentration using the BCA assay kit. Then, 10–30 µg of the protein was separated on a 4%–12% gradient SDS-PAGE with a 1X MOPS running buffer for 1 h at 120 V. Then, the proteins were transferred to PVDF membrane using the iBlot™ 2 Gel Transfer Device (Cat# IB21001, Thermo Fisher Life Technologies Corporation). The membrane was blocked with 5% nonfat milk in 1X Tween 20-Tris buffer saline (TTBS). After 1 h of blocking, the membrane was washed 4 times with agitation of 5 min with 1X TTBS and incubated overnight at 4°C with the primary antibody diluted (anti-CD206 1:1,000 dilution, anti-Arg1 1:1,000 dilution, anti-SR-B1, 1:2000 dilution and anti-β-actin 1:5,000 dilution) in 5% BSA in 1X TTBS. The primary antibody was washed off the membrane four times with 1X TTBS with 5 min agitation and probed with the relevant HRP-linked secondary antibody (all diluted 1:10,000 in 1X TTBS). After a 1 hour-incubation at room temperature, the membrane was washed six times with 1X TTBS with 5 min agitation, and the chemiluminescent bands were detected with the BioRad ChemiDoc MP Imaging System after incubation of the membrane in the Pierce HRP substrate for enhance chemiluminescence and as specified by the manufacturer. The images were saved in a jpeg format and the relative intensity of the bands was acquired using the ImageJ software (https://imagej.nih.gov/, NIH, Bethesda, MD, United States).

#### 2.2.5 Statistical analysis

Unless otherwise stated, all studies were performed at least in three independent replicates. The data were analyzed using the OriginPro 2022b/2023b software (OriginLab Corp., Northampton, MA, United States). Comparisons between two groups were performed using the unpaired two-tailed Student’s t-test. A One-way ANOVA followed by a Tukey’s test was used to evaluate the statistically significant differences in treatment responses when more than two treatment groups were involved in the comparison. The statistical significance was evaluated at *p* < 0.05. The results are presented as mean ± standard deviation (SD).

## 3 Results

### 3.1 B16-F10 CM promotes an M2-like phenotype in RAW 264.7 macrophages

**FIGURE 1:**
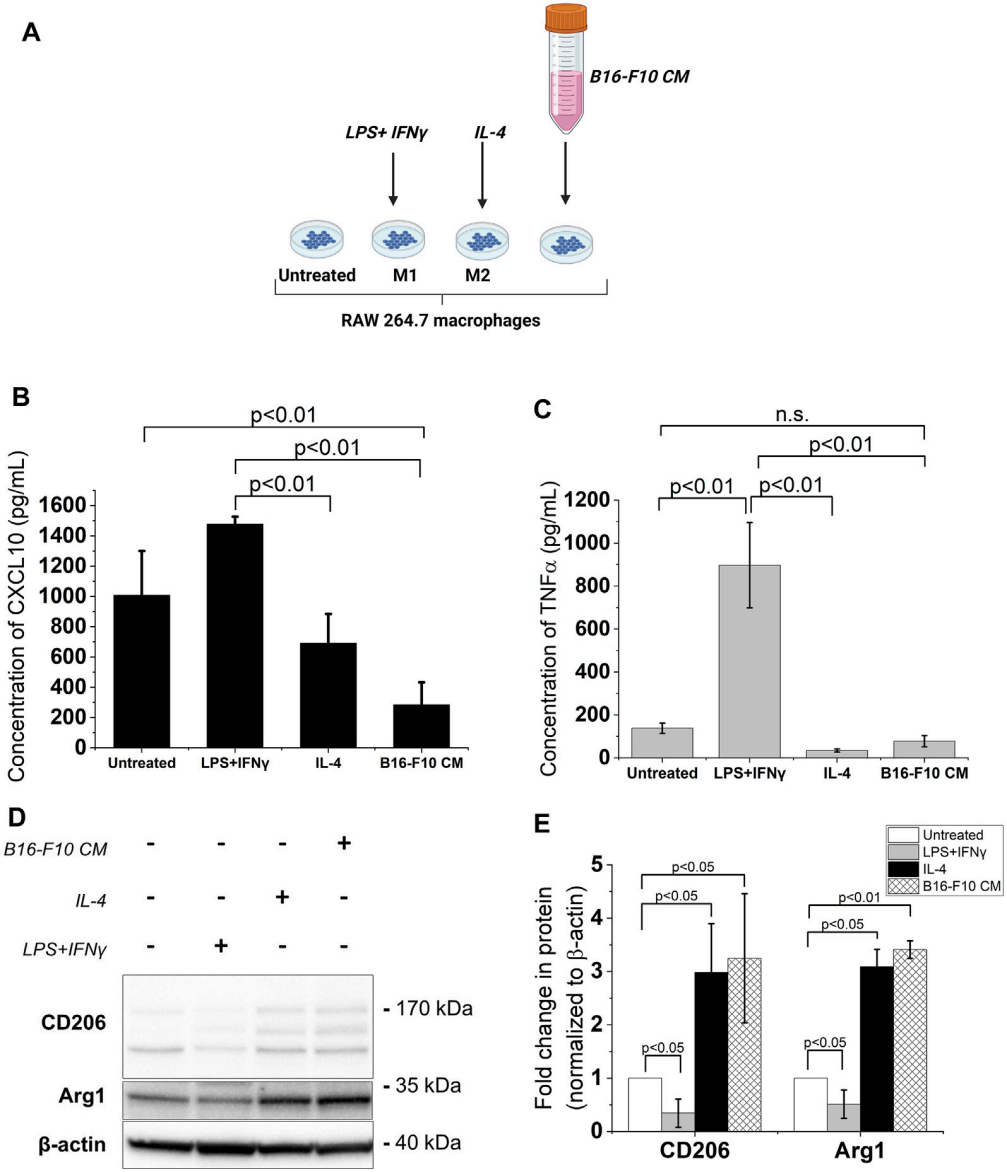
B16-F10 CM treatment increases expression of M2 phenotype markers in RAW 264.7 macrophages. **(A)** The different treatments for RAW 264.7 polarization. Image created with BioRender.com. The macrophages were treated with 50 ng/mL LPS +20 ng/mL IFNγ or 20 ng/mL IL-4 for 24 h. To obtain the B16-F10 CM-educated RAW macrophages, the RAW 264.7 macrophages were incubated in B16-F10-conditioned medium (B16-F10 CM) for 48 h **(B, C)** Cytokine concentrations (assessed via ELISA) of CXCL10 and TNFα in treated RAW 264.7 macrophages compared to untreated RAW 264.7 macrophages. **(D)** Western blot analysis of levels of CD206, CD163 and Arg1 proteins in the treated RAW 264.7 macrophages (a representative for three independent experiments. **(E)** Quantification of protein levels observed in the western blot via ImageJ. All results presented as mean ± SD of at least three independent experiments.

Melanoma cell-derived factors promote an M2-like phenotype in TAMs through their direct or indirect action on these macrophages (Bardi et al., 2018; Di Martile et al., 2020). Hence, in this study, RAW 264.7 murine macrophages were incubated in cDMEM supplemented with the CM from murine melanoma B16-F10 cells to produce an *in vitro* model of TAMs. The LPS + IFNγ-treated RAW 264.7 macrophages served as the M1 reference while the IL-4-treated RAW 264.7 macrophages served as the M2 reference (Mantovani et al., 2004) (Figure 1A). Upon exposure to the B16-F10 CM, secreted levels of CXCL10, an M1 phenotype marker, decreased in RAW 264.7 macrophages (Figure 1B). While treatment with the B16-F10 CM did not induce significant changes in levels of TNFα (also a classical M1 phenotype marker) (Figure 1C), it increased the protein levels of M2 phenotype markers -the mannose receptor CD206 and Arginase 1 (Arg1)- in RAW 264.7 macrophages (Figures 1D,E). These changes in M1 and M2 phenotype markers levels indicate that treatment with B16-F10 CM induces an M2-like phenotype in RAW 264.7 macrophages.

### 3.2 The rHDL-DPM NPs modulate the functional phenotype of B16-F10 CM-educated RAW 264.7 macrophages and enhance DMXAA-induced IFNβ and CXCL10 production

**FIGURE 2:**
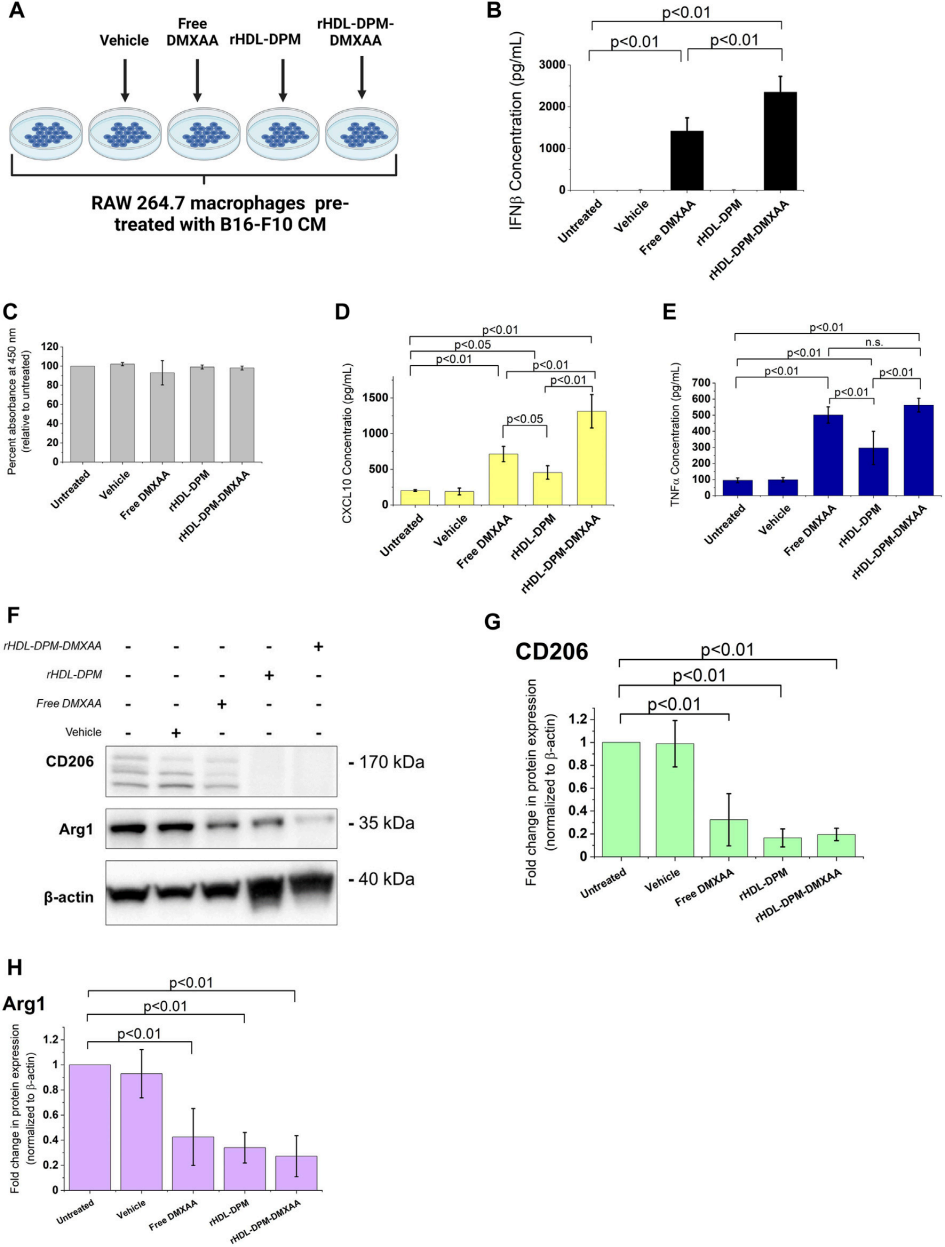
Differential effects of the free DMXAA and rHDL-DPM-DMXAA NPs on B16-F10 CM-educated RAW 264.7 macrophages. **(A)** Treatments and controls of the B16-F10 CM-educated RAW 264.7 macrophages. Image created with BioRender.com. The vehicle is the 7.5% sodium bicarbonate in which the free DMXAA is dissolved. The macrophages were incubated for 24 h with different treatments after which the supernatants were collected for cytokine quantification via ELISA. The cells were collected for protein levels assessment via western blot. **(B)** Concentration of secreted IFNβ in the supernatants of the treated macrophages (assessed via ELISA after treatments). **(C)** Evaluation of cytotoxity on the different treatments on B16-F10 CM-educated RAW 264.7 macrophages. **(D, E)** Concentration of secreted CXCL10 and TNFα in the supernatants of the treated macrophages (assessed via ELISA after treatments, n.s. non-significant). **(F)**. Western blot analysis of the protein levels of CD206, CD163 and Arg1 in the treated B16-F10 CM-educated RAW 264.7 macrophages (a representative blot). **(G–H)**. Quantification of protein levels observed in the western blot via ImageJ. The graphs present results as mean ± SD of four independent experiments.

In line with our previous findings (Dossou et al., 2023), the rHDL-DPM-DMXAA NPs and control formulations exhibited a sub-200 nm z-average of particle diameter size, less than 0.3 in polydispersity index and a negative zeta potential, indicating the formulations are homogeneous and stable (Table 1). No significant differences were observed between rHDL-DPM NPs formulations and their rHDL-DSPE-PEG NPs counterparts. To evaluate changes in the composition of the rHDL-DPM NPs with serum incubation, the ApoA-I was labeled with AF488 and NR was utilized as the payload in the assembly of the nanoparticles to form rHDL(ApoA-I-AF488)-DPM-NR NPs. The fast-protein liquid chromatograph (FPLC) profile coupled with the detection of the nanoparticle components showed that at least 50% of all individual components of the particles, including the payload (NR) co-eluted in the same fraction, suggesting that the integrity of these particles was preserved in the serum (Supplementary Figure S1). As a murine STING agonist, DMXAA can produce an immunostimulatory phenotype in murine M2 macrophages (Downey et al., 2014). Therefore, we hypothesized that the rHDL-DPM-DMXAA NPs would produce an M2-to-M1 phenotype reprogramming in the B16-F10 CM -educated RAW 264.7 macrophages, to the same extent as the free DMXAA. To test this hypothesis, the macrophages were treated with the free DMXAA, the rHDL-DPM DMXAA NPs and their control counterparts (Figure 2A). A hallmark of DMXAA activity is the strong induction of type I interferons, especially IFNβ (Perera et al., 1994; Shirey et al., 2011; Prantner et al., 2012). To confirm the delivery and activity of DMXAA in B16-F10 CM-educated RAW 264.7 macrophages, the levels of secreted IFNβ were evaluated. As expected, the free DMXAA potently induced IFNβ secretion in B16-F10 CM-educated RAW 264.7 macrophages. Interestingly, levels of secreted IFNβ for the DMXAA concentration-matched rHDL-DPM-DMXAA NPs were significantly higher than those elicited by the free DMXAA (Figure 2B). We verified that the different treatments and controls did not have a significant cytotoxic or proliferative effect on the B16-F10 CM-educated macrophages (Figure 2C). This enhancing effect on the M2-to-M1 reprogramming by the mannose-functionalized-rHDL NPs was also observed with levels of secreted CXCL10. In addition, the empty rHDL-DPM NPs also elicited higher levels of CXCL10 (Figure 2D) and TNFα (Figure 2E) compared to the untreated control, although not to the extent of the free DMXAA. However, unlike the case of IFNβ and CXCL10, there was no significant difference in levels of secreted TNFα between the free DMXAA-treated and the rHDL-DPM-DMXAA NPs-treated macrophages. Protein levels of CD206 and Arg1 were also reduced with free DMXAA and rHDL-DPM-DMXAA treatment (Figures 2F–H). Furthermore, the rHDL DPMs also reduced protein levels of CD206 and Arg1 in the macrophages. Overall, these results indicate that, while rHDL-DPM NPs can deliver DMXAA to the B16-F10 CM-educated RAW 264.7 macrophages, they can also modulate macrophage phenotype to enhance the DMXAA-mediated M2-to-M1 phenotype reversal effect.

**TABLE 1:** Characterization of the DMXAA formulations and control formulations. The EE and DL were calculated as described in the method section. N/A: not applicable. *n* = 3. An unpaired Student’s t-test was used to compare values for the rHDL-DPM NPs and their rHDL-DSPE-PEG NPs formulation counterparts. The data are presented as mean ± SD.

Formulations	Particle size (nm)	Polydispersity index	Zeta potential (mV)	EE	DL
rHDL NPs	44.5 ± 6.7	0.238 ± 0.022	−15.8 ± 4.0	N/A	N/A
rHDL-DSPE-PEG NPs	109.8 ± 14.4	0.176 ± 0.014	−24.4 ± 5.1	N/A	N/A
rHDL-DPM NPs	121.5 ± 14.8	0.165 ± 0.007	−25.7 ± 2.3	N/A	N/A
rHDL-DSPE-PEG-DMXAA NPs	101.7 ± 5.6	0.189 ± 0.04	−26.2 ± 5.8	70.7 ± 5.1	2.86 ± 0.34
rHDL-DPM-DMXAA NPs	107.1 ± 4.9	0.195 ± 0.03	−29.9 ± 5.3	73.2 ± 3.2	2.92 ± 0.39

### 3.3 The mannose moiety of the rHDL-DPM NPs enhances the M2-to-M1 phenotype reprogramming of B16-F10 CM-educated RAW 264.7 macrophages

**FIGURE 3:**
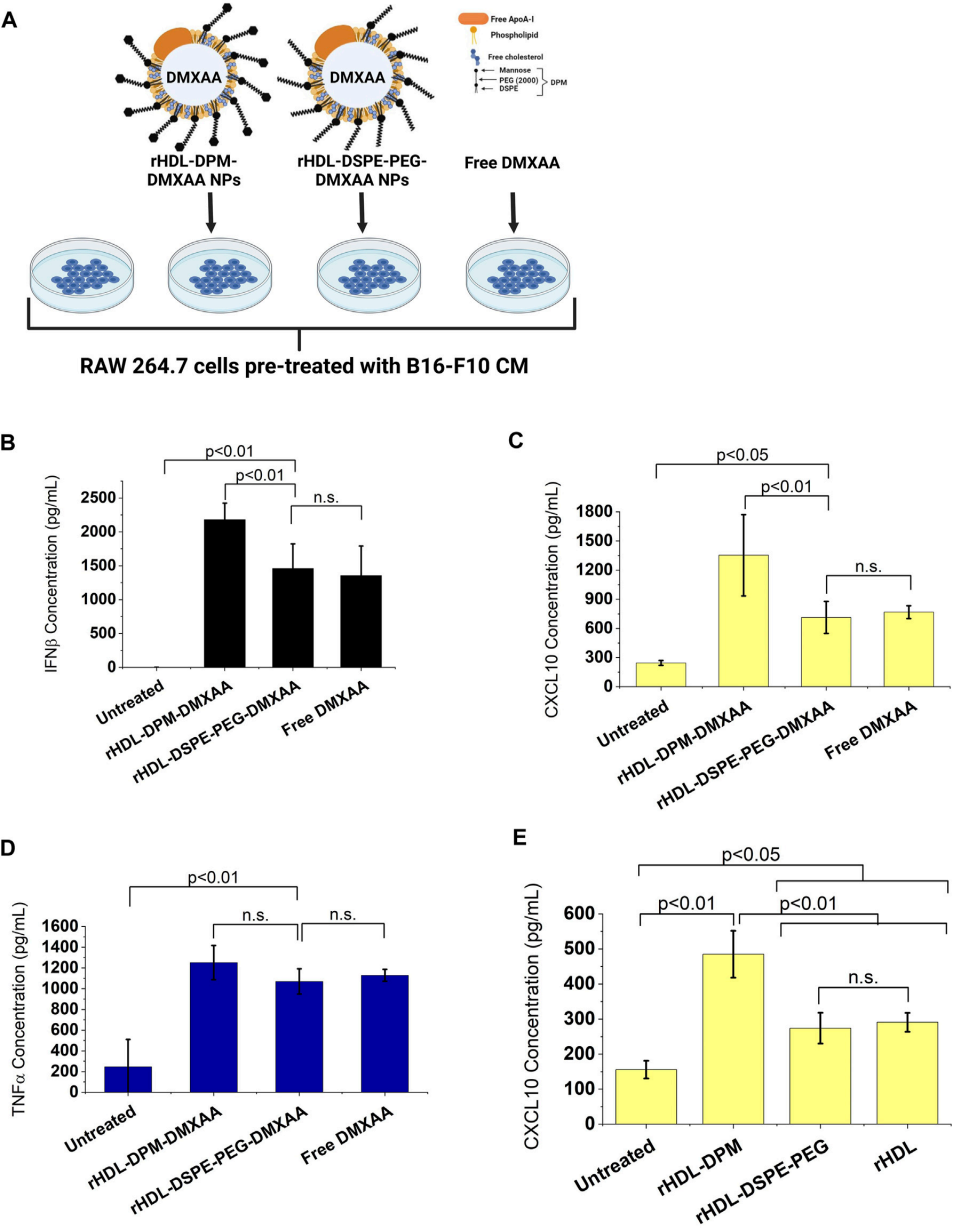
The mannose moiety of the rHDL-DPM NPs enhances the M2-to-M1 reprogramming of B16-F10 CM-educated RAW 264.7 macrophages. **(A)** Schematic illustration of treatments of the B16-F10 CM-educated RAW 264.7 cells with the Free DMXAA, rHDL-DSPE-PEG-DMXAA NPs and rHDL-DPM-DMXAA NPs and untreated control. Image created with BioRender.com. **(B)** Concentration of secreted IFNβ in the supernatants of the treated macrophages. **(C)** Concentration of secreted CXCL10 in the supernatants of the treated macrophages. The absence of mannose abrogates the enhanced immunostimulatory effects of the rHDL-DPM-DMXAA NPs with IFNβ and CXCL10 (n.s., non-significant). **(D)** Concentration of secreted TNFα in the supernatants of the treated macrophages. The lack of mannose did not significantly impact TNFα production **(E)** Effect of the empty nanoparticles on levels secreted CXCL10. Independently of the DPM, the rHDL NPs slightly increase CXCL10 production in the B16-F10 CM -educated RAW 264.7 cells. The cytokine concentrations were determined via ELISA. The results are presented as mean ± SD of three independent experiments.

The addition of mannose moieties to lipid-based NPs transporting M2-to-M1 phenotype reversal agents can enhance the immunostimulatory effect of these formulations on macrophages or dendritic cells (Ye et al., 2020; Zhao et al., 2020; Kim et al., 2021). Moreover, drug-free mannose-decorated liposomes can mediate the M2-to-M1 reversal in macrophages (Ye et al., 2019). In this study, the expected immunostimulatory effects of DMXAA are enhanced when it is delivered to macrophages via the rHDL-DPM NPs. Based on reports indicating the immunostimulatory effects of mannose-functionalization of NPs, we hypothesized that the mannose moiety of the rHDL-DPM NPs contributed to the higher levels of IFNβ and CXCL10 observed with the rHDL-DPM-DMXAA NPs. To test this hypothesis, we utilized rHDL-DSPE-PEG NPs, which lack the terminal mannose, as a carrier for DMXAA (Figure 3A). Levels of IFNβ (Figure 3B) and CXCL10 (Figure 3C) secreted by the B16-F10 CM-educated RAW 264.7 macrophages were lower with the rHDL-DSPE-PEG-DMXAA NPs treatment compared to those from the rHDL-DPM-DMXAA treatment. Additionally, there were no significant differences in the response observed between the rHDL-DSPE-PEG-DMXAA NPs and the free DMXAA. Similarly, there was no significant difference in TNFα produced between treatments with the rHDL-DSPE-PEG-DMXAA NPs and the free DMXAA (Figure 3D). As shown above, the rHDL-DPM NPs increased CXCL10 production in B16-F10 CM-educated RAW 264.7 macrophages. Compared to the rHDL-DPM NPs, both the rHDL-DSPE-PEG NPs and rHDL NPs elicited lower levels of secreted CXCL10 (Figure 3E), indicating that the presence of the mannose contributes to the enhanced CXCL10 production with the rHDL-DPM NPs treatment. However, treatment with DPM alone did not significantly increase CXCL10 secretion (Supplementary Figure S2A), suggesting that complexing DPM to rHDL NPs allows DPM to interact differently with the B16-F10 CM-educated RAW 264.7 macrophages in a way that stimulates production of CXCL10. The CXCL10 levels seen with rHDL-DSPE-PEG NPs treatment were still higher than the untreated B16-F10 CM-educated RAW 264.7 macrophages. The presence of the DSPE-PEG did not significantly contribute to the higher levels of CXCL10 seen with the rHDL-DSPE-PEG NPs compared to the untreated group since there was no significant difference between the effects of the rHDL-DSPE-PEG NPs and the rHDL NPs. To determine which component of the rHDL NPs contributed to the CXCL10 levels, the B16-F10 CM-educated RAW 264.7 macrophages were treated with either the lipid mixture of EYPC-FC and ApoA-I. There were no significant differences between the CXCL10 levels elicited by EYPC-FC mixture and the rHDL NPs, suggesting the EYPC-FC component of the rHDL NPs can stimulate CXCL10 production (Supplementary Figure S2B). Together, these results suggest that the mannose moiety of the rHDL-DPM NPs is the main contributor to the enhanced immunostimulatory effects of the rHDL-DPM-DMXAA NPs, with regards to IFNβ and CXCL10, on the B16-F10 CM-educated RAW 264.7 macrophages.

### 3.4 The rHDL-DPM NPs deliver their payload via the HDL receptor SR-B1 and the mannose receptor CD206

**FIGURE 4:**
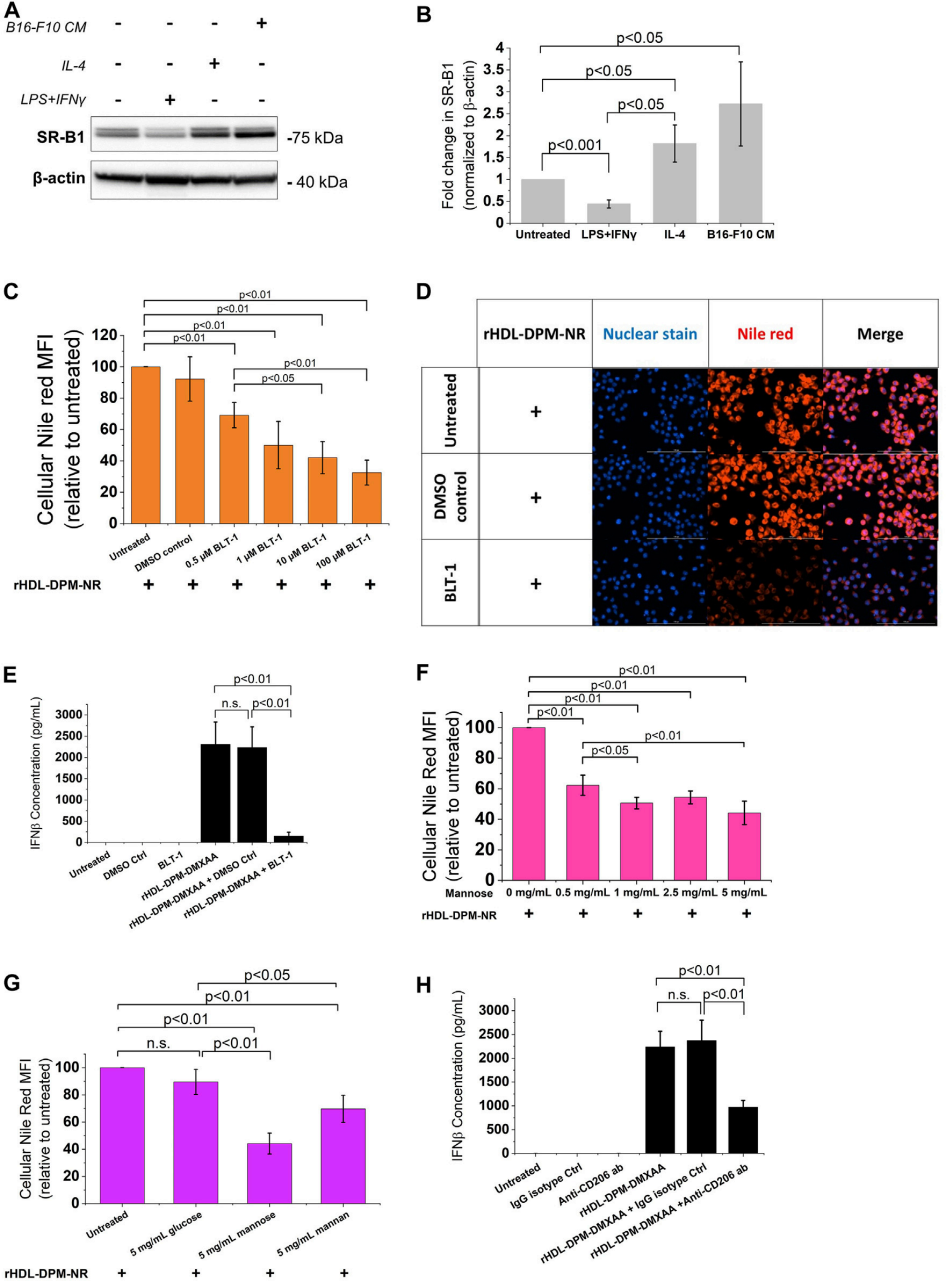
The SR-B1 and CD206 receptors mediate the cargo uptake from rHDL-DPM NPs in B16-F10 CM-educated RAW 264.7 macrophages. **(A)** Representative western blot analysis of RAW 264.7 macrophages treated with LPS + IFNγ, IL-4 and B16-F10 CM. **(B)** Quantification of western blot bands with ImageJ. **(C)** BLT-1 dose-dependent inhibition of NR uptake from rHDL-DPM-NR NPs. BLT-1: Block Lipid Transport-1 (SR-B1 inhibitor). NR: Nile Red. **(D)** Fluorescence microscopy of the effect of BLT-1 (10 µM) pre-treatment on NR uptake when the macrophages are incubated with rHDL-DPM-NR NPs. Scale bar: 100 µm **(E)** Effect of BLT-1 (1 µM) pretreatment on DMXAA-mediated IFNβ production by B16-F10 CM-educated RAW 264 macrophages treated with rHDL-DPM-DMXAA NPs. The cells were treated with BLT-1 for an hour before addition of the rHDL-DPM-DMXAA NPs to allow blockade of the SR-B1 receptor. The levels of IFNβ were determined via ELISA (n.s., non-significant). **(F)** Effect of increasing concentrations of mannose (CD206-mediated uptake inhibitor) on NR uptake. **(G)** Comparison of the effect of mannose, mannan (both CD206-mediated uptake inhibitors) and glucose which does have the same inhibitory activity on CD206. **(H)** Effect of anti-CD206 antibody on DMXAA-mediated IFNβ-production in rHDL-DPM-DMXAA NPs treated cells (assessed via ELISA). Ctrl: control. The data are presented as mean ± SD of three independent experiments.

Several reports show that SR-B1, an HDL receptor, is the major mediator of payload uptake from ApoA-I-based or ApoA-I mimetics-based NPs (Acton et al., 1996; Mooberry et al., 2009; Raut et al., 2018; Zheng et al., 2022). However, the impact of DPM on payload uptake with the rHDL NPs by the cells via SR-B1 is not known. Macrophages express SR-B1 (Ji et al., 2011), and it has been shown that intratumoral TAMs of B16 tumor -bearing mice also express SR-B1 and CD206 (Qian et al., 2017). As discussed above, the B16-F10 CM treatment increased CD206 protein levels in RAW 264.7 macrophages. Western blot analysis showed that treatment with the B16-F10 CM also increased SR-B1 protein levels in RAW 264.7 macrophages (Figures 4A,B). To investigate and visualize the payload uptake, the Nile Red (NR) dye was packaged into the rHDL-DPM NPs. Pre-incubation of B16-F10 CM-educated RAW 264.7 macrophages with increasing concentration of BLT-1 (SR-B1 inhibitor) significantly reduced NR uptake from the rHDL-DPM-NR NPs in a dose-dependent manner, although the uptake was not completely abrogated (Figures 4C,D). Corroborating with the BLT-1-mediated reduction of NR uptake with the rHDL-DPM-NR NPs, pre-treatment with BLT-1 reduced secreted levels of IFNβ in rHDL-DPM-DMXAA NPs-treated macrophages (Figure 4E). The BLT-1 treatment did not elicit IFNβ secretion, nor did it impact the free DMXAA-mediated IFNβ production from the B16-F10 CM-educated RAW 264.7 macrophages (Supplementary Figure S3A). The mannose receptor CD206 has been shown to mediate nanoparticle uptake by macrophages, and is a major contributor to the uptake of mannose-coated NPs (Azad et al., 2014). As the rHDL-DPM NPs are decorated with mannose, we hypothesized that, in addition to SR-B1, CD206 also contributes to the uptake of rHDL-DPM-NR NPs. Mannose and mannan can be utilized to block the uptake activity of CD206 while glucose, which is an epimer of mannose, does not have the same inhibitory capacity on CD206 (Lennartz et al., 1987). The NR uptake from the rHDL-DPM-NPs was significantly diminished following pre-incubation of the macrophages with increasing concentration of mannose (Figure 4F). While similar inhibitory effects on the NR uptake were observed with mannan, pre-incubation with glucose did not significantly affect NR uptake (Figure 4G). Pre-incubation of the B16-F10 CM-educated macrophages with anti-SR-B1 antibody and anti-CD206 antibody also reduced NR uptake from the rHDL-DPM-NR NPs (Supplementary Figure S3B,C). In addition, pre-treatment of the macrophages with anti-CD206 antibody reduced levels of secreted IFNβ in the case of the rHDL-DPM-DMXAA NPs (Figure 4H) but had no significant effect in the case of the Free DMXAA (Supplementary Figure S3D). The uptake of the free NR was not significantly disrupted by the inhibitors (Supplementary Figure S3E–G), and the inhibitors did not cause a significant cytotoxic effect on the B16-F10 CM-educated RAW 264.7 macrophages for the duration of the uptake studies (Supplementary Figure S3H–K). These uptake studies were performed in cDMEM to maintain cell culture conditions used for the rHDL-DPM-DMXAA NPs. The cDMEM contains proteins, including apolipoproteins which have been reported to make up a substantial part of the protein corona surrounding NPs (Bros et al., 2018; Elechalawar et al., 2020). To confirm that SR-B1 and CD206 mediate the uptake independently from the serum proteins potentially surrounding the rHDL-DPM-NR NPs, the cells were incubated in DMEM without FBS. Pre-treatment with BLT-1 and mannose still reduced NR uptake from the rHDL-DPM-NR NPs although the BLT-1 had a more pronounced inhibitory effect in the cDMEM condition (Supplementary Figure S3L). Taken together, these results confirm the contribution of both the SR-B1 and CD206 in the payload uptake from the rHDL-DPM NPs.

### 3.5 The ApoA-I and the mannose moiety of the rHDL-DPM NPs mediate cellular payload uptake via SR-B1 and CD206 through endocytic and non-endocytic mechanisms

**FIGURE 5:**
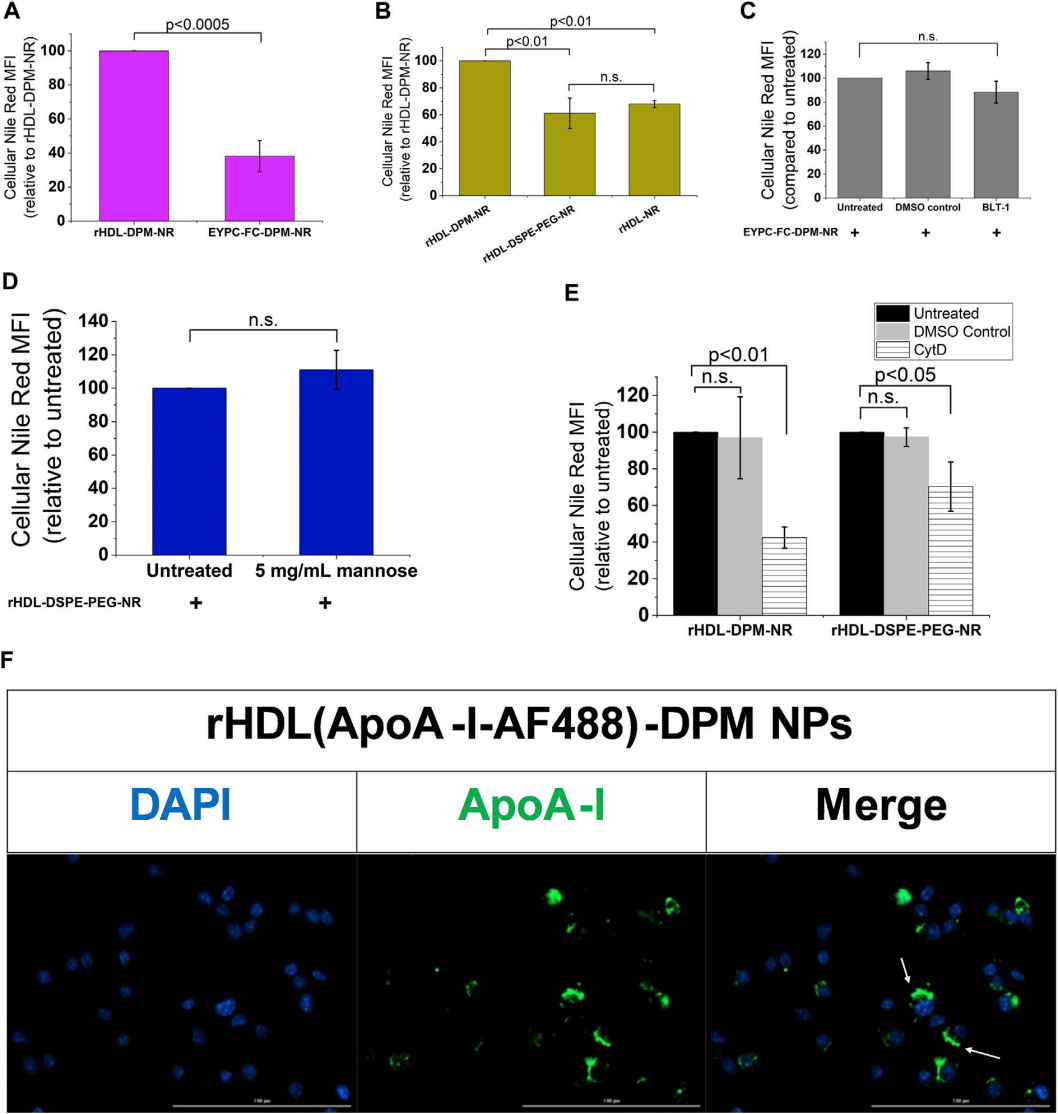
The ApoA-I/SR-B1 and the mannose moiety/CD206 interactions mediate cellular NR uptake through endocytic and non-endocytic mechanisms. **(A)** Cellular NR uptake comparison with B16-F10 CM-educated RAW 264.7 macrophages incubated either with the rHDL-DPM- NR and the EYPC-FC-DPM-NR (no ApoA-I). NR: Nile Red. **(B)** Effect of absence of mannose and DSPE-PEG on cellular NR uptake. **(C)** Effect of BLT-1 (10 µM) on cellular NR uptake in the absence of ApoA-I (n.s., non-significant). **(D)** Effect of mannose (5 mg/mL) -mediated CD206 inhibition on cellular NR uptake in the absence of the mannose moiety. **(E)** Effect of CytD (5 μg/mL)-mediated endocytosis inhibition on cellular NR uptake. **(F)** Fluorescence microscopy on the B16-F10 CM -educated RAW 264.7 macrophages incubated with the rHDL(ApoA-I-AF488)-DPM NPs. Scale bar: 100 µm. The white arrows point to the AF488-labeled ApoA-I near the plasma membrane. The results are presented as mean ± SD of three independent experiments.

While mannosylated objects can interact with CD206 (van der Zande et al., 2021), it has been demonstrated that ApoA-I interacts with SR-B1 at the plasma membrane to mediate the intracellular uptake of HDL particles or the content of these particles (de Beer et al., 2001; Liu et al., 2002; Powers and Sahoo, 2022). However, besides ApoA-I, SR-B1 recognizes several other ligands, including PEG (Shen et al., 2018; Su et al., 2021) which is one of the components of the rHDL-DPM NPs. To confirm the contribution of ApoA-I and the mannose moiety in the NR uptake, various formulations of NR were prepared and found to exhibit similar physical characteristics as those discussed above for the DMXAA formulations. These include the sub-200 nm hydrodynamic diameter size of the particles (Supplementary Figure S34A,B), a polydispersity index of less than 0.3 (Supplementary Figure S4C) and a negative zeta potential (Supplementary Figure S4D). Incubation of the B16-F10 CM-educated RAW 264.7 macrophages with the EYPC-FC-DPM-NR NPs, which contains no ApoA-I, resulted in lower cellular NR uptake when compared with the rHDL-DPM-NR NPs (Figure 5A). Also, the cellular NR uptake was significantly diminished in rHDL-DSPE-PEG-NR NPs and the rHDL-NR NPs both of which lack the mannose moiety, although there was no significant difference in cellular NR uptake between the rHDL-DSPE-PEG-NR NPs and rHDL-NR NPs (Figure 5B). BLT- 1 did not have significant inhibitory effect on cellular NR uptake from the EYPC-FC-DPM-NR NPs (Figure 5C), indicating that the SR-B1 does not significantly contribute to the cellular NR uptake in the absence of ApoA-I. Pre-treatment with mannose did not significantly prevent the NR uptake by the cells from the rHDL-DSPE-PEG-NR NPs, also suggesting that the mannose moiety is important for the involvement of the mannose receptor in the cellular NR uptake (Figure 5D). While reports show that CD206 mediates uptake of particles via endocytosis (van der Zande et al., 2021), both endocytic and non-endocytic mechanisms have been noted for the SR-B1- mediated uptake of HDL contents or extracellular objects (Silver et al., 2001; Nieland et al., 2005; Pagler et al., 2006; Zhang et al., 2019; Plochberger et al., 2020; Taban et al., 2023). To investigate the payload uptake mechanism, the macrophages were pre-treated with cytochalasin D (CytD), an actin polymerization inhibitor which targets macropinocytosis, phagocytosis, clathrin-mediated endocytosis and clathrin-independent endocytosis (Francia et al., 2019). CytD reduced cellular NR uptake with the rHDL-DPM-NR NPs but did not completely abrogate it (Figure 5E). Of note, the CytD-mediated cellular NR uptake inhibition was less pronounced in the case of the rHDL-DSPE-PEG-NR NPs than in the case of the rHDL-DPM-NR NPs. No significant cytotoxic effect was observed with CytD treatment on the B16-F10 CM-educated RAW 264.7 macrophages (Supplementary Figure S4E). Additionally, cellular NR uptake involving the free NR was not significantly impacted by pretreatment with CytD (Supplementary Figure S4F). These findings suggest that the ApoA-I/SR-B1-mediated cellular NR uptake relies on mechanisms other than the ones inhibited by CytD. Incubation of the macrophages with rHDL-DPM NPs where ApoA-I is labeled with AF488 revealed that, while some ApoA-I can be found intracellularly like the NR, most of it is located at the plasma membrane (Figure 5F). To ensure that the AF488 did not impact on the ability of ApoA-I to bind to lipids, thereby impacting the localization of ApoA-I in these uptake studies, a comparison of steady-state fluorescence anisotropy of rHDL(ApoA-I-AF488) and that of the free AF488 and ApoA-I-AF488 was conducted as previously described (Shah et al., 2016; Dossou et al., 2023). The increasing anisotropy of AF488 suggests that the ApoA-I-AF488 is associated with the lipids (Supplementary Figure S5A). In addition, the FPLC profile shows that the ApoA-I-AF488 co-elutes with other nanoparticles components including phospholipids and DPM (Supplementary Figure S5B,D). The labeling of the nanoparticles significantly increased the hydrodynamic size of the particles (Supplementary Figure S5D), the PDI (Supplementary Figure S5E) and slightly reduced the zeta potential (Supplementary Figure S5F). Together, these results indicate that the combination of ApoA-I/SR-B1 and the mannose moiety/CD206 interactions mediates the cellular NR uptake through endocytic and non-endocytic mechanisms.

### 3.6 The rHDL-DPM NPs are more effective at delivering payload to macrophages than to the B16-F10 melanoma cells

**FIGURE 6:**
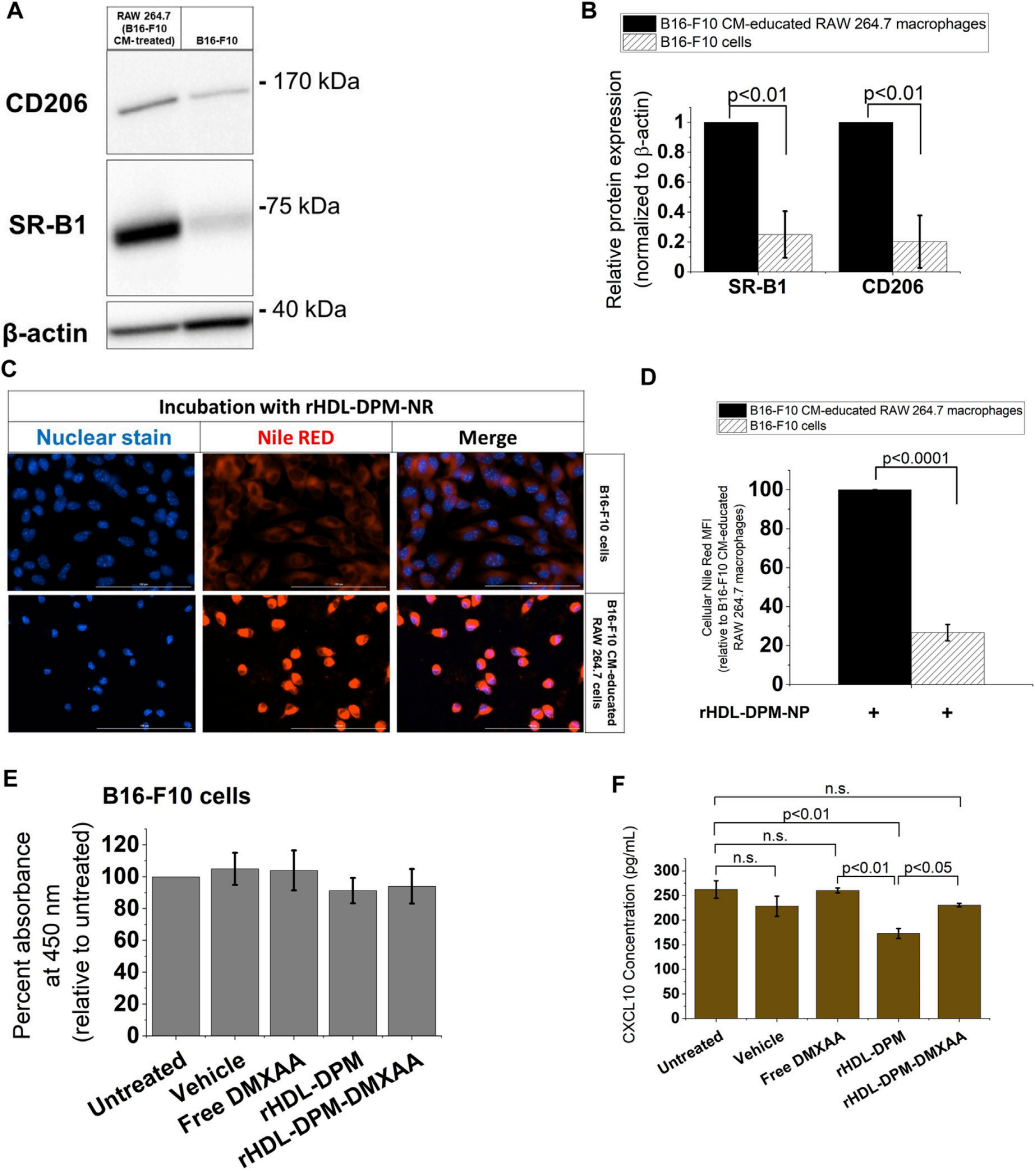
The rHDL-DPM NPs deliver payload more effectively to the B16-F10 CM-educated RAW 264.7 macrophages than the B16-F10 cells. **(A)** A representative western blot analysis of SR-B1 and CD206 expression of B16-F10 CM-educated RAW 264.7 macrophages and the B16-F10 cells. **(B)** Quantification of bands in **(A)** via ImageJ. **(C)** Relative cellular NR uptake of the B16-F10 CM-educated RAW 264.7 macrophages and B16-F10 CM. **(D)** Fluorescence microscopy of cellular NR uptake. Scale bar: 100 µm. **(E)** Assessment of cytotoxic effect of vehicle, Free DMXAA, rHDL-DPM NPs, rHDL-DPM-DMXAA NPs on B16-F10 cells. **(F)** Quantification of secreted CXCL10 via ELISA. The data are presented as mean ± SD of three independent experiments.

As the rHDL-DPM NPs are being evaluated in their ability to target B16-F10 CM-educated RAW 264.7 macrophages, their impact on the cancer cells remains to be explored. In western blot analyses, B16-F10 cells show negligible protein levels of CD206 as well as lower SR-B1 expression compared to the B16-F10 CM-educated RAW 264.7 macrophages (Figures 6A,B). Accordingly, less NR uptake was observed with the B16-F10 cells compared to the B16-F10 CM-educated RAW 264.7 macrophages following incubation with the rHDL-DPM-NR NPs (Figures 6C,D). However, since there is some level of payload uptake in the B16-F10 cells, we sought to investigate the modulation of cytokine production in the B16-F10 cells by the rHDL-DPM NPs. There was no significant cytotoxic effect from the vehicle, Free DMXAA, rHDL-DPM NPs and rHDL-DPM-DMXAA NPs on the B16-F10 cells (Figure 6E). In contrast to their immunostimulatory effect on the B16-F10 CM-educated RAW 264.7 macrophages, there was no detectable levels of secreted IFNβ or TNFα from the B16-F10 cells following treatment with the Free DMXAA, rHDL-DPM-DMXAA NPs and associated controls (data not shown). Interestingly, treatment with rHDL-DPM NPs slightly reduced CXCL10 production in the B16-F10 cells while the Free DMXAA did not have a significant effect on levels of secreted CXCL10 (Figure 6F). These results indicate that the rHDL-DPM NPs are more targeted to the B16-F10 CM educated RAW 264.7 macrophages than to the B16-F10 cells. In addition, they underscore the differential response of the B16-F10 cells and the macrophages to both the Free DMXAA and the rHDL-DPM NPs.

### 3.7 The rHDL-DPM NPs can impact B16-F10 proliferation and sensitivity to PTX by modulating macrophages secretory phenotype

**FIGURE 7:**
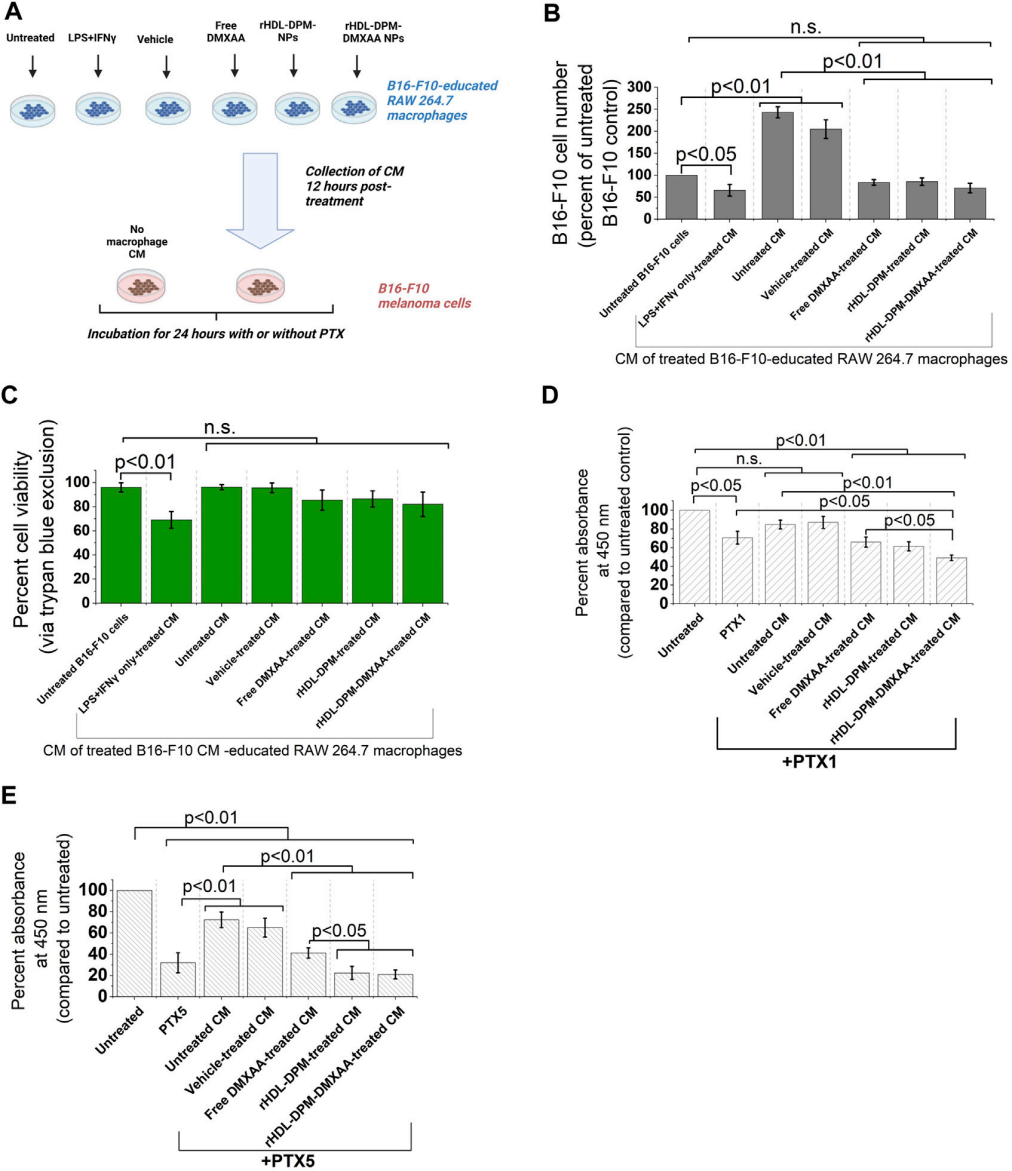
The rHDL-DPM NPs can impact B16-F10 cell proliferation and sensitivity to PTX by modulating the secretory phenotype of macrophages **(A)** Workflow of B16-F10 cell treatment with macrophages CM. CM: conditioned medium. Image created with BioRender.com. **(B)** Cell number (percent of untreated control) recovered after incubation of the B16-F10 cells with the different CM (n.s., non-significant). **(C)** Cell viability via trypan blue exclusion assay after incubation of B16-F10 cells with the CM resulting from the different treatments of the macrophages. **(D)** Cytotoxic effect (via CCK8) of PTX1 (paclitaxel, 1 μg/mL) on B16-F10 cells exposed to the different macrophage CM. **(E)** Cytotoxic effect of PTX5 (paclitaxel, 5 μg/mL) on B16-F10 cells exposed to the different macrophage CM. The data are presented as mean ± SD of at least three independent replicates.

The functional phenotype of TAMs can impact tumor progression, including cancer cell proliferation (Chen et al., 2019). In melanoma, secreted products from M2-like TAMs can enhance cancer cell proliferation, survival, and can contribute to chemoresistance of melanoma cells to anticancer agents (Tham et al., 2014; Pieniazek et al., 2018; Shi et al., 2022). Conversely, it has been demonstrated in other cancer types that conditioned media from M1 (LPS + IFNγ -treated) macrophages can inhibit cancer cell proliferation (Engstrom et al., 2014; Song et al., 2018). In light of the enhanced M2-to-M1 phenotype reversal mediated by the rHDL-DPM-DMXAA NPs in the macrophages, we asked whether the conditioned media from rHDL-DPM-DMXAA NPs-treated RAW 264.7 macrophages could affect the proliferation of B16-F10 cells proliferation and their response to PTX, a classic chemotherapeutic agent used for advanced melanoma (Luke and Schwartz, 2013). To investigate this question, B16-F10 cells were exposed to conditioned media from treated RAW 264.7 macrophages (Figure 7A). In accordance with the anti-proliferative effects on cancer cells described for M1 macrophages, the LPS + IFNγ-treated B16-F10 CM-educated RAW 264.7 CM reduced cell number and cell viability (assessed via trypan blue) in the B16-F10 cell culture. Unlike their untreated CM counterpart, the CM from Free DMXAA-, rHDL-DPM NPs- or rHDL-DPM-DMXAA NPs-treated macrophages does not enhance B16-F10 cell proliferation (Figure 7B). The cell viability of B16-F10 cells was not affected by these treatments (Figure 7C). To discern and evaluate the impact of the treated macrophages CM on PTX-mediated cytotoxicity, we used low doses of PTX: 1 μg/mL (PTX1) and 5 μg/mL (PTX5). While the CM from the untreated macrophages diminished the cytotoxic effect of PTX on B16-F10 cells, the CM from the Free DMXAA-, rHDL-DPM NPs- and the rHDL-DPM-DMXAA NPs-treated macrophages improved the cytotoxic effect of PTX (PTX1 and PTX5) on B16-F10 cells. Furthermore, compared with the Free DMXAA, the rHDL-DPM-DMXAA NPs treatment of macrophages produced greater cytotoxicity with PTX1 (Figure 7D) and PTX5 (Figure 7E). Interestingly, the rHDL-DPM NPs also produced a greater cytotoxicity when compared with the Free DMXAA treatment of macrophages with PTX5. Collectively, these findings suggest that the rHDL-DPM NPs can curb B16-F10 proliferation and potentiate PTX-mediated cell death by modulating the secretory phenotype of the B16-F10 CM-educated RAW 264.7 macrophages.

## 4 Discussion

One of the factors impeding the sustained effectiveness of immunotherapies in the treatment of advanced melanoma is the highly immunosuppressive TME which does not facilitate the intratumoral infiltration and activity of cytotoxic immune cells (Sambi et al., 2019; Dobosz et al., 2022). TAMs account for up to 30% of the melanoma tumor content (Hussein, 2006) and are polarized to an M2-like, immunosuppressive phenotype by melanoma cell-derived factors including but not limited to the B-cell lymphoma 2 protein (Di Martile et al., 2020), acidosis (Vitale et al., 2019), colony-stimulating factor 1, CXCL12 and CCL2 (Pieniazek et al., 2018). Herein, the RAW 264.7 murine macrophages were exposed to CM from B16-F10 murine melanoma cells, which contains secreted factors from the cancer cells, as a reductionist *in vitro* approach to obtain melanoma TAMs. The ensuing increase in protein levels of M2 immunosuppressive phenotype markers (CD206 and Arg1) and the decrease in the M1 phenotype marker CXCL10 observed in this study indicate that the B16-F10 CM was able to educate the RAW 264.7 macrophages to an M2-like, immunosuppressive phenotype. These results align with clinical observation of the M2-polarizing effect of cancer cells on intratumoral macrophages during the progression of the disease (Falleni et al., 2017).

Owing to their abundance in the TME, their plasticity and key role in modulating the innate and adaptive arms of the immune system, TAMs offer a unique gateway to addressing immunosuppression in the TME Several approaches, including STING agonism have been devised to limit their substantial contribution to immunosuppression and to reprogram them to enable anticancer immune activation (Li et al., 2022). However, the ubiquity of macrophages in the body (Epelman et al., 2014) calls for the selective delivery of immunomodulatory agents, including STING agonists, to TAMs in the tumor to improve the efficacy of these agents and to avoid adverse immune effects. In this study, we evaluated the ability of the mannose-functionalized rHDL NPs or rHDL-DPM NPs to serve as a delivery vehicle to the B16-F10 CM-educated RAW 264.7 macrophages. Unlike the empty rHDL-DPM NPs, the assembled rHDL-DPM-DMXAA NPs were able to elicit IFNβ production from the B16-F10 CM-educated RAW 264.7 macrophages, indicating that the DMXAA was delivered to the B16-F10 CM-educated RAW 264.7 macrophages and retained its type I interferon -inducing activity. Importantly, the M2 markers CD206, and Arg1 protein levels were reduced while the M1 phenotype markers CXCL10 and TNFα were increased by treatment with rHDL-DPM-DMXAA NPs. These results confirmed that an M2-to-M1 phenotype reversal was facilitated by the rHDL-DPM-DMXAA NPs in the B16-F10 CM-educated RAW 264.7 macrophages.

The modulation of these M2 markers is clinically relevant in melanoma treatment. It has been demonstrated that the mannose receptor CD206 on TAMs can dampen T-cell activation by supporting upregulation of CTLA-4 and CD45 inhibition on T cells (Schuette et al., 2016). Arg1 is associated with immunosuppression, and the reduction of the TAM population exhibiting these markers improves prognosis (Arlauckas et al., 2018). While acute ApoA-I exposure has been reported to promote the M1 phenotype, including increased CXCL10 expression in TAMs in B16-F10 tumor-bearing mice (Zamanian-Daryoush et al., 2013), we did not observe a significant change in secreted levels of CXCL10 or TNFα (data not shown) with the direct exposure of the B16-F10 CM-educated RAW 264.7 macrophages to ApoA-I. This may be due to a dose-dependent effect of ApoA-I. In addition, ApoA-I has been shown to alter cancer cell metabolism (Zamanian-Daryoush et al., 2020), and this may indirectly mediate the M1 phenotype observed in TAMs when tumor-bearing mice are administered ApoA-I. Thus, the negligible ApoA-I effect observed on the B16-F10 CM-educated RAW 264.7 macrophages could also be due to limitations of the *in vitro* model used in this study.

The rHDL-DPM-DMXAA NPs provided the most benefit with the M1 cytokines assessed. As described for the Free DMXAA (Ching et al., 1999; Cao et al., 2001; Shirey et al., 2011), treatment of the B16-F10 CM-educated RAW 264.7 with the rHDL-DPM-DMXAA NPs increased IFNβ, CXCL10 and TNFα secretion. Furthermore, the mannose moiety on the rHDL-DPM-DMXAA NPs significantly enhanced secretion of IFNβ and CXCL10 compared to the effects of the Free DMXAA. This immunostimulatory effect of the mannose moiety has been reported in other drug delivery platforms (Ye et al., 2019; Kim et al., 2021; Bellato et al., 2022) and could be a result of the recognition of the NPs and subsequent activation of the mannose receptor CD206 (Milone and Fitzgerald-Bocarsly, 1998; Jaynes et al., 2020). The absence of significant enhancement in secreted TNFα with the rHDL-DPM-DMXAA NPs compared to the Free DMXAA could be due to a ceiling effect where a maximum amount of TNFα is already being induced by the Free DMXAA treatment. The rHDL-DPM NPs also decreased CD206 and Arg1 protein levels. This modulation of CD206 and Arg1 protein levels by mannose-decorated NPs was also reported by Ye et al. (Ye et al., 2019; Glass et al., 2022), and provides an additional avenue to addressing immunosuppression. Differential effects were found with treatment of the B16-F10 cells with rHDL-DPM-DMXAA NPs which did not elicit significant IFNβ or TNFα production. These results are in agreement with the findings that the CD11b+ cells in the TME, which would predominantly identify macrophages, are the major contributor to pro-inflammatory cytokine production including TNFα when lung cancer-bearing mice were treated with DMXAA (Jassar et al., 2005). Moreover, the suppression of the cGAS-STING pathway found in cancer cells (Suter et al., 2021) could be another factor leading to the lack of production of the M1 phenotype-related cytokines in the B16-F10 cells upon treatment with the DMXAA formulations.

In an *in vivo* setting, the increased levels of CXCL10 and IFNβ observed with the rHDL-DPM-DMXAA NPs may be beneficial in promoting antitumor effects when combined with other therapeutic approaches. It has been reported that CXCL10 is important for the efficacy of immune checkpoint inhibitors (House et al., 2020). As an adjuvant therapy modality in melanoma, the administration of IFNβ in the clinics enhances relapse-free survival (Davar et al., 2012). As well, the combination of IFNβ administration with immune checkpoint inhibitors or targeted therapies sustains tumor regression (Litvin et al., 2015; Audsley et al., 2021). Moreover, IFNβ modulates TAMs phenotype towards an M1 phenotype (Kakizaki et al., 2015) and can sensitize cancer cells to chemotherapy (Makita et al., 2019). While chemotherapy is not the first-line treatment for advanced melanoma, it is often used after immunotherapy/targeted therapy failure in patients, and chemoresistance is one of the barriers to its effectiveness (American Cancer Society, 2022b; Goldinger et al., 2022; Kalal et al., 2017). PTX is part of the chemotherapy portfolio used in advanced melanoma (Samoylenko et al., 2016; Maeda et al., 2022), and its effectiveness can be dampened by various drug resistance mechanisms (Castro et al., 2022). The ability of the CM from the rHDL-DPM NPs-, and rHDL-DPM-DMXAA NPs-treated macrophages to modulate the proliferation and to improve the sensitivity of the B16-F10 cells to PTX suggest that the rHDL-DPM NPs could be a valuable tool in mitigating chemoresistance to PTX in B16-F10 tumor-bearing mice.

In addition to confirming the immunomodulatory effects of the rHDL-DPM-DMXAA NPs on the macrophages and their indirect effect on the B16-F10 cells, we also uncovered major players in payload uptake from the rHDL-DPM NPs. In line with reports on high expression of SR-B1 on TAMs (Qian et al., 2017; Xiong et al., 2023) the B16-F10 CM increased SR-B1 protein levels in RAW 264.7 macrophages. The uptake studies showed that both SR-B1 and CD206 mediate payload delivery to the macrophages, with ApoA-I likely interacting with SR-B1 and the mannose being recognized by CD206 at the plasma membrane. Endocytic and non-endocytic mechanisms mediated the uptake of the NR with non-endocytic mechanisms being primarily attributed to SR-B1 since NPs lacking the mannose moiety were less affected by the blocking of endocytosis. The importance of ApoA-I/SR-B1 and mannose/CD206 in mediating payload delivery provides guidance for enhanced design of these NPs as delivery agents. It also provides a blueprint to determine cells that would be most susceptible to rHDL-DPM NPs-mediated delivery. Hence, the B16-F10 cells, which have lower expression of SR-B1 and CD206, uptake less payload than the B16-F10 CM-educated RAW 264.7 macrophages. Notably, studies have demonstrated that HDL-type NPs preferentially deliver payload to M2-like TAMs compared to other cells present in the TME (Perez-Medina et al., 2015; Dong et al., 2023; Xiong et al., 2023). The addition of the mannose moiety to the rHDL NPs enhanced NR uptake in the B16-F10 CM-educated RAW 264.7 macrophages, suggesting that the rHDL-DPM NPs have the potential to improve TAM targeting in B16-F10 *in vivo* models. Complete abrogation of the NR uptake could not be achieved, and this is likely due to a combination of nanoparticle surface loading of NR, leakage of free NR from the NPs, and the internalization of the rHDL-DPM-NPs by other minor mechanisms. While the PEG did not significantly contribute to the SR-B1-mediated uptake, its presence seemingly did not hinder it as Pedersbæk et al. have also shown (Pedersbæk et al., 2020).

While these findings on the effects of rHDL-DPM-DMXAA NPs confirmed our hypothesis, there are some limitations to the study design and considerations in the generalization of findings regarding the rHDL-DPM NPs. First, the 2D *in vitro* cell culture does not account for biological barriers-tissues, fluids, the tumor architecture-that often impact on nanoparticle homing and payload delivery efficiency (Anchordoquy et al., 2017; Pedersbaek et al., 2019; Kopac, 2021). While the *in vivo* assessment of the rHDL-DPM NPs and of the effect of these barriers is beyond the scope of this study, we have found SR-B1 and CD206 were still relevant in payload uptake whether the rHDL-DPM NPs were incubated in serum-free or cDMEM conditions which suggests that the protein corona does not significantly impede payload delivery mechanism. Second, the use of conditioned media of cancer cells and macrophages does not account for the direct physical interaction between macrophages and cancer cells as well as the influence of other cell types on macrophage phenotype and cancer cell behavior. In addition, the RAW 264.7 macrophages are a leukemic cell line, and they do not recapitulate all features of mouse or human primary macrophages. For the scope of our study, the effect of B16-F10 CM on M2 and M1 markers in the RAW 264.7 macrophages echoes to some extent the M2-like immunosuppressive behavior described for TAMs in melanoma. Thus, they are still useful in these preliminary investigations of the M2-to-M1 phenotype reversal paradigm. Third, SR-B1 and CD206 are both expressed by the liver and dendritic cells (Vasquez et al., 2017; Shen et al., 2018), suggesting that the liver and dendritic cells in the TME may be potential targets for the rHDL-DPM NPs. It has been demonstrated that compared to other lipid-based particles, payloads from HDL-type particles tend to accumulate more efficiently in cancerous tissue while other major homing tissues are the liver and the kidney (Niora et al., 2020; Pedersbaek and Simonsen, 2020). Hence, *in vivo* studies in B16-F10 tumor-bearing mice are warranted to evaluate the biodistribution and safety of the rHDL-DPM NPs, the impact of the empty and drug-loaded rHDL-DPM NPs on tumor progression, on the sensitivity of the tumor to PTX and on the different types of cells in the TME. As well, *in vivo* studies would be important to determine any significant impact on the tumor from the biological activity of the increased production of IFNβ and CXCL10 levels elicited by the rHDL-DPM-DMXAA NPs compared to the Free DMXAA.

Despite these limitations, the findings from the present study clearly demonstrate that addition of the mannose moiety to the rHDL NPs improves payload delivery to the B16-F10 CM-educated RAW 264.7 macrophages via SR-B1 and CD206. They also show that the rHDL-DPM NPs can reprogram the B16-F10 CM-educated RAW 264.7 macrophages to an M1 phenotype and that NPs-treated macrophages can increase sensitivity to PTX in B16-F10 melanoma cells. Based on these findings, we anticipate that the rHDL-DPM NPs are likely to improve TAM targeting and treatment outcome when combined with immunotherapy or PTX *in vivo* B16-F10 mouse models or *in vivo* models of cancers where the pro-tumoral M2-TAMs exhibit high SR-B1 and CD206 expression.

## Data Availability

The raw data supporting the conclusion of this article will be made available by the authors, without undue reservation. Further inquiries should be directed to akpedjedossou@my.unthsc.edu or to nirupama.sabnis@unthsc.edu.

## References

[B1] Acton S. Rigotti A. Landschulz K. T. Xu S. Hobbs H. H. Krieger M. (1996). Identification of scavenger receptor SR-BI as a high density lipoprotein receptor. Science 271 (5248), 518–520. 10.1126/science.271.5248.518 8560269

[B2] American Cancer Society (2023a). Cancer facts and figures 2023. Atlanta, GA: American Cancer Society. Available from: https://www.cancer.org/content/dam/cancer-org/research/cancer-facts-and-statistics/annual-cancer-facts-and-figures/2023/2023-cancer-facts-and-figures.pdf .

[B3] American Cancer Society (2022b). Treatment of melanoma skin cancer. Stage Atlanta, GA: American Cancer Society. Available from: https://www.cancer.org/cancer/types/melanoma-skin-cancer/treating/by-stage.html (Accessed March 1, 2022).

[B4] Anchordoquy T. J. Barenholz Y. Boraschi D. Chorny M. Decuzzi P. Dobrovolskaia M. A. (2017). Mechanisms and barriers in cancer nanomedicine: addressing challenges, looking for solutions. ACS Nano 11 (1), 12–18. 10.1021/acsnano.6b08244 28068099 PMC5542883

[B5] Arlauckas S. P. Garren S. B. Garris C. S. Kohler R. H. Oh J. Pittet M. J. (2018). Arg1 expression defines immunosuppressive subsets of tumor-associated macrophages. Theranostics 8 (21), 5842–5854. 10.7150/thno.26888 30613266 PMC6299430

[B6] Audsley K. M. Wagner T. Ta C. Newnes H. V. Buzzai A. C. Barnes S. A. (2021). IFNβ is a potent adjuvant for cancer vaccination strategies. Front. Immunol. 12, 735133. 10.3389/fimmu.2021.735133 34552594 PMC8450325

[B7] Azad A. K. Rajaram M. V. Schlesinger L. S. (2014). Exploitation of the macrophage mannose receptor (CD206) in infectious disease diagnostics and therapeutics. J. Cytol. Mol. Biol. 1 (1), 1000003. 10.13188/2325-4653.1000003 24672807 PMC3963702

[B8] Bardi G. T. Smith M. A. Hood J. L. (2018). Melanoma exosomes promote mixed M1 and M2 macrophage polarization. Cytokine 105, 63–72. 10.1016/j.cyto.2018.02.002 29459345 PMC5857255

[B9] Bellato F. Feola S. Dalla Verde G. Bellio G. Pirazzini M. Salmaso S. (2022). Mannosylated polycations target CD206(+) antigen-presenting cells and mediate T-cell-specific activation in cancer vaccination. Biomacromolecules 23 (12), 5148–5163. 10.1021/acs.biomac.2c00993 36394394 PMC9748946

[B10] Bonaventura P. Shekarian T. Alcazer V. Valladeau-Guilemond J. Valsesia-Wittmann S. Amigorena S. (2019). Cold tumors: a therapeutic challenge for immunotherapy. Front. Immunol. 10, 168. 10.3389/fimmu.2019.00168 30800125 PMC6376112

[B11] Bros M. Nuhn L. Simon J. Moll L. Mailander V. Landfester K. (2018). The protein corona as a confounding variable of nanoparticle-mediated targeted vaccine delivery. Front. Immunol. 9, 1760. 10.3389/fimmu.2018.01760 30116246 PMC6082927

[B12] Cao Z. Baguley B. C. Ching L. M. (2001). Interferon-inducible protein 10 induction and inhibition of angiogenesis *in vivo* by the antitumor agent 5,6-dimethylxanthenone-4-acetic acid (DMXAA). Cancer Res. 61 (4), 1517–1521. 11245459

[B13] Castro M. V. Barbero G. A. Mascolo P. Ramos R. Quezada M. J. Lopez-Bergami P. (2022). ROR2 increases the chemoresistance of melanoma by regulating p53 and Bcl2-family proteins via ERK hyperactivation. Cell. Mol. Biol. Lett. 27 (1), 23. 10.1186/s11658-022-00327-7 35260073 PMC8903712

[B14] Ceci C. Atzori M. G. Lacal P. M. Graziani G. (2020). Targeting tumor-associated macrophages to increase the efficacy of immune checkpoint inhibitors: a glimpse into novel therapeutic approaches for metastatic melanoma. Cancers (Basel) 12 (11), 3401. 10.3390/cancers12113401 33212945 PMC7698460

[B15] Chelvanambi M. Fecek R. J. Taylor J. L. Storkus W. J. (2021). STING agonist-based treatment promotes vascular normalization and tertiary lymphoid structure formation in the therapeutic melanoma microenvironment. J. Immunother. Cancer 9 (2), e001906. 10.1136/jitc-2020-001906 33526609 PMC7852948

[B16] Chen Y. Song Y. Du W. Gong L. Chang H. Zou Z. (2019). Tumor-associated macrophages: an accomplice in solid tumor progression. J. Biomed. Sci. 26 (1), 78. 10.1186/s12929-019-0568-z 31629410 PMC6800990

[B17] Ching L. M. Goldsmith D. Joseph W. R. Korner H. Sedgwick J. D. Baguley B. C. (1999). Induction of intratumoral tumor necrosis factor (TNF) synthesis and hemorrhagic necrosis by 5,6-dimethylxanthenone-4-acetic acid (DMXAA) in TNF knockout mice. Cancer Res. 59 (14), 3304–3307. 10416582

[B18] Chipurupalli S. Ganesan R. Dhanabal S. P. Kumar M. S. Robinson N. (2020). Pharmacological STING activation is a potential alternative to overcome drug-resistance in melanoma. Front. Oncol. 10, 758. 10.3389/fonc.2020.00758 32477956 PMC7241280

[B19] Chong Y. P. Peter E. P. Lee F. J. M. Chan C. M. Chai S. Ling L. P. C. (2022). Conditioned media of pancreatic cancer cells and pancreatic stellate cells induce myeloid-derived suppressor cells differentiation and lymphocytes suppression. Sci. Rep. 12 (1), 12315. 10.1038/s41598-022-16671-9 35853996 PMC9296552

[B20] Chung T. W. Chung C. H. Lue Y. F. (2000). A colorimetric method for determining distearoylphosphatidylethanolamine-polyethylene glycol 2000 in blood suspension. Anal. Biochem. 285 (2), 264–267. 10.1006/abio.2000.4733 11017712

[B21] CiRen B. Wang X. Long Z. (2016). The evaluation of immunotherapy and chemotherapy treatment on melanoma: a network meta-analysis. Oncotarget 7 (49), 81493–81511. 10.18632/oncotarget.13277 27845904 PMC5348408

[B22] Corrales L. Glickman L. H. McWhirter S. M. Kanne D. B. Sivick K. E. Katibah G. E. (2015). Direct activation of STING in the tumor microenvironment leads to potent and systemic tumor regression and immunity. Cell. Rep. 11 (7), 1018–1030. 10.1016/j.celrep.2015.04.031 25959818 PMC4440852

[B23] Davar D. Tarhini A. A. Kirkwood J. M. (2012). Adjuvant therapy for melanoma. Cancer J. 18 (2), 192–202. 10.1097/PPO.0b013e31824f118b 22453021 PMC3478763

[B24] de Beer M. C. Durbin D. M. Cai L. Jonas A. de Beer F. C. van der Westhuyzen D. R. (2001). Apolipoprotein A-I conformation markedly influences HDL interaction with scavenger receptor BI. J. Lipid Res. 42 (2), 309–313. 10.1016/s0022-2275(20)31693-x 11181762

[B25] Di Martile M. Farini V. Consonni F. M. Trisciuoglio D. Desideri M. Valentini E. (2020). Melanoma-specific bcl-2 promotes a protumoral M2-like phenotype by tumor-associated macrophages. J. Immunother. Cancer 8 (1), e000489. 10.1136/jitc-2019-000489 32269145 PMC7254128

[B26] Dobosz P. Stepien M. Golke A. Dzieciatkowski T. (2022). Challenges of the immunotherapy: perspectives and limitations of the immune checkpoint inhibitor treatment. Int. J. Mol. Sci. 23 (5), 2847. 10.3390/ijms23052847 35269988 PMC8910928

[B27] Dong Q. Han D. Li B. Yang Y. Ren L. Xiao T. (2023). Bionic lipoprotein loaded with chloroquine-mediated blocking immune escape improves antitumor immunotherapy. Int. J. Biol. Macromol. 240, 124342. 10.1016/j.ijbiomac.2023.124342 37030459

[B28] Dossou A. S. Mantsch M. E. Kapic A. Burnett W. L. Sabnis N. Coffer J. L. (2023). Mannose-coated reconstituted lipoprotein nanoparticles for the targeting of tumor-associated macrophages: optimization, characterization, and *in vitro* evaluation of effectiveness. Pharmaceutics 15 (6), 1685. 10.3390/pharmaceutics15061685 37376134 PMC10304188

[B29] Downey C. M. Aghaei M. Schwendener R. A. Jirik F. R. (2014). DMXAA causes tumor site-specific vascular disruption in murine non-small cell lung cancer, and like the endogenous non-canonical cyclic dinucleotide STING agonist, 2'3'-cGAMP, induces M2 macrophage repolarization. PLoS One 9 (6), e99988. 10.1371/journal.pone.0099988 24940883 PMC4062468

[B30] Elechalawar C. K. Hossen M. N. McNally L. Bhattacharya R. Mukherjee P. (2020). Analysing the nanoparticle-protein corona for potential molecular target identification. J. Control Release 322, 122–136. 10.1016/j.jconrel.2020.03.008 32165239 PMC7675788

[B31] Engstrom A. Erlandsson A. Delbro D. Wijkander J. (2014). Conditioned media from macrophages of M1, but not M2 phenotype, inhibit the proliferation of the colon cancer cell lines HT-29 and CACO-2. Int. J. Oncol. 44 (2), 385–392. 10.3892/ijo.2013.2203 24296981 PMC3898868

[B32] Epelman S. Lavine K. J. Randolph G. J. (2014). Origin and functions of tissue macrophages. Immunity 41 (1), 21–35. 10.1016/j.immuni.2014.06.013 25035951 PMC4470379

[B33] Falleni M. Savi F. Tosi D. Agape E. Cerri A. Moneghini L. (2017). M1 and M2 macrophages’ clinicopathological significance in cutaneous melanoma. Melanoma Res. 27 (3), 200–210. 10.1097/CMR.0000000000000352 28272106

[B34] Frampton A. E. Sivakumar S. (2022). A new combination immunotherapy in advanced melanoma. N. Engl. J. Med. 386 (1), 91–92. 10.1056/NEJMe2116892 34986291

[B35] Francia V. Reker-Smit C. Boel G. Salvati A. (2019). Limits and challenges in using transport inhibitors to characterize how nano-sized drug carriers enter cells. Nanomedicine (Lond) 14 (12), 1533–1549. 10.2217/nnm-2018-0446 31208280

[B36] Glass E. B. Hoover A. A. Bullock K. K. Madden M. Z. Reinfeld B. I. Harris W. (2022). Stimulating TAM-mediated anti-tumor immunity with mannose-decorated nanoparticles in ovarian cancer. BMC Cancer 22 (1), 497. 10.1186/s12885-022-09612-2 35513776 PMC9074180

[B37] Goldinger S. M. Buder-Bakhaya K. Lo S. N. Forschner A. McKean M. Zimmer L. (2022). Chemotherapy after immune checkpoint inhibitor failure in metastatic melanoma: a retrospective multicentre analysis. Eur. J. Cancer 162, 22–33. 10.1016/j.ejca.2021.11.022 34952480

[B38] Gorshkova I. N. Mei X. Atkinson D. (2014). Binding of human apoA-I[K107del] variant to TG-rich particles: implications for mechanisms underlying hypertriglyceridemia. J. Lipid Res. 55 (9), 1876–1885. 10.1194/jlr.M047241 24919401 PMC4617355

[B39] He Y. de Araujo Junior R. F. Cruz L. J. Eich C. (2021). Functionalized nanoparticles targeting tumor-associated macrophages as cancer therapy. Pharmaceutics 13 (10), 1670. 10.3390/pharmaceutics13101670 34683963 PMC8540805

[B40] House I. G. Savas P. Lai J. Chen A. X. Y. Oliver A. J. Teo Z. L. (2020). Macrophage-derived CXCL9 and CXCL10 are required for antitumor immune responses following immune checkpoint blockade. Clin. Cancer Res. 26 (2), 487–504. 10.1158/1078-0432.CCR-19-1868 31636098

[B41] Hussein M. R. (2006). Tumour-associated macrophages and melanoma tumourigenesis: integrating the complexity. Int. J. Exp. Pathol. 87 (3), 163–176. 10.1111/j.1365-2613.2006.00478.x 16709225 PMC2517364

[B42] Hwang J. Zheng M. Wiraja C. Cui M. Yang L. Xu C. (2020). Reprogramming of macrophages with macrophage cell membrane-derived nanoghosts. Nanoscale Adv. 2 (11), 5254–5262. 10.1039/d0na00572j 36132036 PMC9419214

[B43] Jassar A. S. Suzuki E. Kapoor V. Sun J. Silverberg M. B. Cheung L. (2005). Activation of tumor-associated macrophages by the vascular disrupting agent 5,6-dimethylxanthenone-4-acetic acid induces an effective CD8+ T-cell-mediated antitumor immune response in murine models of lung cancer and mesothelioma. Cancer Res. 65 (24), 11752–11761. 10.1158/0008-5472.CAN-05-1658 16357188

[B44] Jaynes J. M. Sable R. Ronzetti M. Bautista W. Knotts Z. Abisoye-Ogunniyan A. (2020). Mannose receptor (CD206) activation in tumor-associated macrophages enhances adaptive and innate antitumor immune responses. Sci. Transl. Med. 12 (530), eaax6337. 10.1126/scitranslmed.aax6337 32051227 PMC7832040

[B45] Ji A. Meyer J. M. Cai L. Akinmusire A. de Beer M. C. Webb N. R. (2011). Scavenger receptor SR-BI in macrophage lipid metabolism. Atherosclerosis 217 (1), 106–112. 10.1016/j.atherosclerosis.2011.03.017 21481393 PMC3139003

[B46] Kakizaki A. Fujimura T. Furudate S. Kambayashi Y. Yamauchi T. Yagita H. (2015). Immunomodulatory effect of peritumorally administered interferon-beta on melanoma through tumor-associated macrophages. Oncoimmunology 4 (11), e1047584. 10.1080/2162402X.2015.1047584 26451326 PMC4589056

[B47] Kalal B. S. Upadhya D. Pai V. R. (2017). Chemotherapy resistance mechanisms in advanced skin cancer. Oncol. Rev. 11 (1), 326. 10.4081/oncol.2017.326 28382191 PMC5379221

[B48] Kato M. Neil T. K. Fearnley D. B. McLellan A. D. Vuckovic S. Hart D. N. (2000). Expression of multilectin receptors and comparative FITC-dextran uptake by human dendritic cells. Int. Immunol. 12 (11), 1511–1519. 10.1093/intimm/12.11.1511 11058570

[B49] Kim D. Wu Y. Shim G. Oh Y. K. (2021). Lipid nanoparticle-mediated lymphatic delivery of immunostimulatory nucleic acids. Pharmaceutics 13 (4), 490. 10.3390/pharmaceutics13040490 33916667 PMC8103501

[B50] Kopac T. (2021). Protein corona, understanding the nanoparticle-protein interactions and future perspectives: a critical review. Int. J. Biol. Macromol. 169, 290–301. 10.1016/j.ijbiomac.2020.12.108 33340622

[B51] Koyama M. Tanaka M. Dhanasekaran P. Lund-Katz S. Phillips M. C. Saito H. (2009). Interaction between the N- and C-terminal domains modulates the stability and lipid binding of apolipoprotein A-I. Biochemistry 48 (11), 2529–2537. 10.1021/bi802317v 19239199 PMC2936823

[B52] Lennartz M. R. Wileman T. E. Stahl P. D. (1987). Isolation and characterization of a mannose-specific endocytosis receptor from rabbit alveolar macrophages. Biochem. J. 245 (3), 705–711. 10.1042/bj2450705 3663187 PMC1148189

[B53] Li M. He L. Zhu J. Zhang P. Liang S. (2022). Targeting tumor-associated macrophages for cancer treatment. Cell. Biosci. 12 (1), 85. 10.1186/s13578-022-00823-5 35672862 PMC9172100

[B54] Litvin O. Schwartz S. Wan Z. Schild T. Rocco M. Oh N. L. (2015). Interferon α/β enhances the cytotoxic response of MEK inhibition in melanoma. Mol. Cell. 57 (5), 784–796. 10.1016/j.molcel.2014.12.030 25684207 PMC4355234

[B55] Liu C. Y. Xu J. Y. Shi X. Y. Huang W. Ruan T. Y. Xie P. (2013). M2-polarized tumor-associated macrophages promoted epithelial-mesenchymal transition in pancreatic cancer cells, partially through TLR4/IL-10 signaling pathway. Lab. Invest. 93 (7), 844–854. 10.1038/labinvest.2013.69 23752129

[B56] Liu T. Krieger M. Kan H. Y. Zannis V. I. (2002). The effects of mutations in helices 4 and 6 of ApoA-I on scavenger receptor class B type I (SR-BI)-mediated cholesterol efflux suggest that formation of a productive complex between reconstituted high density lipoprotein and SR-BI is required for efficient lipid transport. J. Biol. Chem. 277 (24), 21576–21584. 10.1074/jbc.M112103200 11882653

[B57] Luke J. J. Schwartz G. K. (2013). Chemotherapy in the management of advanced cutaneous malignant melanoma. Clin. Dermatol 31 (3), 290–297. 10.1016/j.clindermatol.2012.08.016 23608448 PMC3709980

[B58] Ma X. Song Q. Gao X. (2018). Reconstituted high-density lipoproteins: novel biomimetic nanocarriers for drug delivery. Acta Pharm. Sin. B 8 (1), 51–63. 10.1016/j.apsb.2017.11.006 29872622 PMC5985628

[B59] MacCuaig W. M. McNally L. R. (2020). Development of an (89)Zr-labeled high-density lipoprotein nanoparticle as a PET agent to track efficacy of immunotherapy. Radiol. Imaging Cancer 2 (3), e204018. 10.1148/rycan.2020204018 33778720 PMC7983789

[B60] Maeda T. Hiura A. Uehara J. Toyoshima R. Nakagawa T. Yoshino K. (2022). Combined carboplatin and paclitaxel therapy improves overall survival in patients with nivolumab-resistant acral and mucosal melanoma. Br. J. Dermatol 186 (2), 361–363. 10.1111/bjd.20758 34510408

[B61] Makita K. Hara H. Sano E. Okamoto Y. Ochiai Y. Harada T. (2019). Interferon-beta sensitizes human malignant melanoma cells to temozolomide-induced apoptosis and autophagy. Int. J. Oncol. 54 (5), 1864–1874. 10.3892/ijo.2019.4743 30864696

[B62] Mantovani A. Sica A. Sozzani S. Allavena P. Vecchi A. Locati M. (2004). The chemokine system in diverse forms of macrophage activation and polarization. Trends Immunol. 25 (12), 677–686. 10.1016/j.it.2004.09.015 15530839

[B63] Marabelle A. Andtbacka R. Harrington K. Melero I. Leidner R. de Baere T. (2018). Starting the fight in the tumor: expert recommendations for the development of human intratumoral immunotherapy (HIT-IT). Ann. Oncol. 29 (11), 2163–2174. 10.1093/annonc/mdy423 30295695 PMC6290929

[B64] Mei Y. Tang L. Xiao Q. Zhang Z. Zhang Z. Zang J. (2021). Reconstituted high density lipoprotein (rHDL), a versatile drug delivery nanoplatform for tumor targeted therapy. J. Mater Chem. B 9 (3), 612–633. 10.1039/d0tb02139c 33306079

[B65] Meric-Bernstam F. Sweis R. F. Hodi F. S. Messersmith W. A. Andtbacka R. H. I. Ingham M. (2022). Phase I dose-escalation trial of MIW815 (ADU-S100), an intratumoral STING agonist, in patients with advanced/metastatic solid tumors or lymphomas. Clin. Cancer Res. 28 (4), 677–688. 10.1158/1078-0432.CCR-21-1963 34716197

[B66] Milone M. C. Fitzgerald-Bocarsly P. (1998). The mannose receptor mediates induction of IFN-alpha in peripheral blood dendritic cells by enveloped RNA and DNA viruses. J. Immunol. 161 (5), 2391–2399. 10.4049/jimmunol.161.5.2391 9725235

[B67] Mooberry L. K. Nair M. Paranjape S. McConathy W. J. Lacko A. G. (2009). Receptor mediated uptake of paclitaxel from a synthetic high density lipoprotein nanocarrier. J. Drug Target. 00 (00), 090728052632043–090728052632046. 10.1080/10611860903156419 19637935

[B68] Nieland T. J. Ehrlich M. Krieger M. Kirchhausen T. (2005). Endocytosis is not required for the selective lipid uptake mediated by murine SR-BI. Biochim. Biophys. Acta 1734 (1), 44–51. 10.1016/j.bbalip.2005.02.007 15866482

[B69] Nieland T. J. Shaw J. T. Jaipuri F. A. Duffner J. L. Koehler A. N. Banakos S. (2008). Identification of the molecular target of small molecule inhibitors of HDL receptor SR-BI activity. Biochemistry 47 (1), 460–472. 10.1021/bi701277x 18067275 PMC2736594

[B70] Niora M. Pedersbaek D. Munter R. Weywadt M. F. V. Farhangibarooji Y. Andresen T. L. (2020). Head-to-Head comparison of the penetration efficiency of lipid-based nanoparticles into tumor spheroids. ACS Omega 5 (33), 21162–21171. 10.1021/acsomega.0c02879 32875252 PMC7450641

[B72] Pagler T. A. Rhode S. Neuhofer A. Laggner H. Strobl W. Hinterndorfer C. (2006). SR-BI-mediated high density lipoprotein (HDL) endocytosis leads to HDL resecretion facilitating cholesterol efflux. J. Biol. Chem. 281 (16), 11193–11204. 10.1074/jbc.M510261200 16488891

[B73] Pedersbaek D. Kraemer M. K. Kempen P. J. Ashley J. Braesch-Andersen S. Andresen T. L. (2019). The composition of reconstituted high-density lipoproteins (rHDL) dictates the degree of rHDL cargo- and size-remodeling via direct interactions with endogenous lipoproteins. Bioconjug Chem. 30 (10), 2634–2646. 10.1021/acs.bioconjchem.9b00552 31487985

[B74] Pedersbaek D. Simonsen J. B. (2020). A systematic review of the biodistribution of biomimetic high-density lipoproteins in mice. J. Control Release 328, 792–804. 10.1016/j.jconrel.2020.09.038 32971201

[B75] Pedersbæk D. Krogager L. Albertsen C. H. Ringgaard L. Hansen A. E. Jønsson K. (2020). Effect of apoA-I PEGylation on the biological fate of biomimetic high-density lipoproteins. ACS Omega 6, 871–880. 10.1021/acsomega.0c05468 33458538 PMC7808163

[B76] Perera P. Y. Barber S. A. Ching L. M. Vogel S. N. (1994). Activation of LPS-inducible genes by the antitumor agent 5,6-dimethylxanthenone-4-acetic acid in primary murine macrophages. Dissection of signaling pathways leading to gene induction and tyrosine phosphorylation. J. Immunol. 153 (10), 4684–4693. 10.4049/jimmunol.153.10.4684 7525711

[B77] Perez-Medina C. Tang J. Abdel-Atti D. Hogstad B. Merad M. Fisher E. A. (2015). PET imaging of tumor-associated macrophages with 89Zr-labeled high-density lipoprotein nanoparticles. J. Nucl. Med. 56 (8), 1272–1277. 10.2967/jnumed.115.158956 26112022 PMC4737475

[B78] Pieniazek M. Matkowski R. Donizy P. (2018). Macrophages in skin melanoma-the key element in melanomagenesis. Oncol. Lett. 15 (4), 5399–5404. 10.3892/ol.2018.8021 29552183 PMC5840697

[B79] Plochberger B. Sych T. Weber F. Novacek J. Axmann M. Stangl H. (2020). Lipoprotein particles interact with membranes and transfer their cargo without receptors. Biochemistry 59 (45), 4421–4428. 10.1021/acs.biochem.0c00748 33147967 PMC7677925

[B80] Powers H. R. Sahoo D. (2022). SR-B1's next top model: structural perspectives on the functions of the HDL receptor. Curr. Atheroscler. Rep. 24 (4), 277–288. 10.1007/s11883-022-01001-1 35107765 PMC8809234

[B81] Prantner D. Perkins D. J. Lai W. Williams M. S. Sharma S. Fitzgerald K. A. (2012). 5,6-Dimethylxanthenone-4-acetic acid (DMXAA) activates stimulator of interferon gene (STING)-dependent innate immune pathways and is regulated by mitochondrial membrane potential. J. Biol. Chem. 287 (47), 39776–39788. 10.1074/jbc.M112.382986 23027866 PMC3501038

[B82] Qian Y. Qiao S. Dai Y. Xu G. Dai B. Lu L. (2017). Molecular-targeted immunotherapeutic strategy for melanoma via dual-targeting nanoparticles delivering small interfering RNA to tumor-associated macrophages. ACS Nano 11 (9), 9536–9549. 10.1021/acsnano.7b05465 28858473

[B83] Raut S. Mooberry L. Sabnis N. Garud A. Dossou A. S. Lacko A. (2018). Reconstituted HDL: drug delivery platform for overcoming biological barriers to cancer therapy. Front. Pharmacol. 9, 1154. 10.3389/fphar.2018.01154 30374303 PMC6196266

[B84] Sambi M. Bagheri L. Szewczuk M. R. (2019). Current challenges in cancer immunotherapy: multimodal approaches to improve efficacy and patient response rates. J. Oncol. 2019, 4508794. 10.1155/2019/4508794 30941175 PMC6420990

[B85] Samoylenko I. Kharkevich G. Petenko N. N. Orlova K. V. Sinelnikov I. Utyashev I. A. (2016). Paclitaxel and carboplatin chemotherapy in patients with metaststic melanoma refractory to BRAF/MEK inhibitors. J. Clin. Oncol. 34 (15), 9552. 10.1200/jco.2016.34.15_suppl.9552

[B86] Schuette V. Embgenbroich M. Ulas T. Welz M. Schulte-Schrepping J. Draffehn A. M. (2016). Mannose receptor induces T-cell tolerance via inhibition of CD45 and up-regulation of CTLA-4. Proc. Natl. Acad. Sci. U. S. A. 113 (38), 10649–10654. 10.1073/pnas.1605885113 27601670 PMC5035904

[B87] Shah S. Chib R. Raut S. Bermudez J. Sabnis N. Duggal D. (2016). Photophysical characterization of anticancer drug valrubicin in rHDL nanoparticles and its use as an imaging agent. J. Photochem Photobiol. B 155, 60–65. 10.1016/j.jphotobiol.2015.12.007 26735001 PMC5531042

[B88] Shen W. J. Azhar S. Kraemer F. B. (2018). SR-B1: a unique multifunctional receptor for cholesterol influx and efflux. Annu. Rev. Physiol. 80, 95–116. 10.1146/annurev-physiol-021317-121550 29125794 PMC6376870

[B89] Shi Q. Shen Q. Liu Y. Shi Y. Huang W. Wang X. (2022). Increased glucose metabolism in TAMs fuels O-GlcNAcylation of lysosomal Cathepsin B to promote cancer metastasis and chemoresistance. Cancer Cell. 40 (10), 1207–1222.e10. 10.1016/j.ccell.2022.08.012 36084651

[B90] Shirey K. A. Nhu Q. M. Yim K. C. Roberts Z. J. Teijaro J. R. Farber D. L. (2011). The anti-tumor agent, 5,6-dimethylxanthenone-4-acetic acid (DMXAA), induces IFN-beta-mediated antiviral activity *in vitro* and *in vivo* . J. Leukoc. Biol. 89 (3), 351–357. 10.1189/jlb.0410216 21084628 PMC3040469

[B91] Silver D. L. Wang N. Xiao X. Tall A. R. (2001). High density lipoprotein (HDL) particle uptake mediated by scavenger receptor class B type 1 results in selective sorting of HDL cholesterol from protein and polarized cholesterol secretion. J. Biol. Chem. 276 (27), 25287–25293. 10.1074/jbc.M101726200 11301333

[B92] Simonsen J. B. (2016). Evaluation of reconstituted high-density lipoprotein (rHDL) as a drug delivery platform - a detailed survey of rHDL particles ranging from biophysical properties to clinical implications. Nanomedicine 12 (7), 2161–2179. 10.1016/j.nano.2016.05.009 27237620

[B93] Song W. Thakor P. Vesey D. A. Gobe G. C. Morais C. (2018). Conditioned medium from stimulated macrophages inhibits growth but induces an inflammatory phenotype in breast cancer cells. Biomed. Pharmacother. 106, 247–254. 10.1016/j.biopha.2018.06.126 29966967

[B94] Strober W. (2015). Trypan blue exclusion test of cell viability. Curr. Protoc. Immunol. 111, A3–B3. 10.1002/0471142735.ima03bs111 PMC6716531 26529666

[B95] Su X. Ramirez-Escudero M. Sun F. van den Dikkenberg J. B. van Steenbergen M. J. Pieters R. J. (2021). Internalization and transport of PEGylated lipid-based mixed micelles across caco-2 cells mediated by scavenger receptor B1. Pharmaceutics 13 (12), 2022. 10.3390/pharmaceutics13122022 34959304 PMC8703698

[B96] Sun L. Li Z. Shang H. Xin X. (2021). Hypericin enhances paclitaxel-induced B16-F10 cell apoptosis by activating a cytochrome c release-dependent pathway. Front. Pharmacol. 12, 652452. 10.3389/fphar.2021.652452 34421585 PMC8371448

[B97] Sundararajan S. Thida A. M. Yadlapati S. (2022). Metastatic melanoma. Treasure Island, FL: StatPearls. 29262232

[B98] Suter M. A. Tan N. Y. Thiam C. H. Khatoo M. MacAry P. A. Angeli V. (2021). cGAS-STING cytosolic DNA sensing pathway is suppressed by JAK2-STAT3 in tumor cells. Sci. Rep. 11 (1), 7243. 10.1038/s41598-021-86644-x 33790360 PMC8012641

[B99] Taban Q. Ahmad S. M. Mumtaz P. T. Bhat B. Haq E. Magray S. (2023). Scavenger receptor B1 facilitates the endocytosis of *Escherichia coli* via TLR4 signaling in mammary gland infection. Cell. Commun. Signal 21 (1), 3. 10.1186/s12964-022-01014-y 36604713 PMC9813905

[B100] Tardif J. C. Gregoire J. L'Allier P. L. Ibrahim R. Lesperance J. Heinonen T. M. (2007). Effects of reconstituted high-density lipoprotein infusions on coronary atherosclerosis: a randomized controlled trial. JAMA 297 (15), 1675–1682. 10.1001/jama.297.15.jpc70004 17387133

[B101] Tham M. Tan K. W. Keeble J. Wang X. Hubert S. Barron L. (2014). Melanoma-initiating cells exploit M2 macrophage TGFβ and arginase pathway for survival and proliferation. Oncotarget 5 (23), 12027–12042. 10.18632/oncotarget.2482 25294815 PMC4322977

[B102] van der Zande H. J. P. Nitsche D. Schlautmann L. Guigas B. Burgdorf S. (2021). The mannose receptor: from endocytic receptor and biomarker to regulator of (Meta)Inflammation. Front. Immunol. 12, 765034. 10.3389/fimmu.2021.765034 34721436 PMC8551360

[B103] Vasquez M. Simoes I. Consuegra-Fernandez M. Aranda F. Lozano F. Berraondo P. (2017). Exploiting scavenger receptors in cancer immunotherapy: lessons from CD5 and SR-B1. Eur. J. Immunol. 47 (7), 1108–1118. 10.1002/eji.201646903 28504304

[B104] Vitale I. Manic G. Coussens L. M. Kroemer G. Galluzzi L. (2019). Macrophages and metabolism in the tumor microenvironment. Cell. Metab. 30 (1), 36–50. 10.1016/j.cmet.2019.06.001 31269428

[B105] Wang J. Zheng C. Zhai Y. Cai Y. Lee R. J. Xing J. (2021). High-density lipoprotein modulates tumor-associated macrophage for chemoimmunotherapy of hepatocellular carcinoma. Nano Today 37, 101064. 10.1016/j.nantod.2020.101064

[B106] Wang Q. Bergholz J. S. Ding L. Lin Z. Kabraji S. K. Hughes M. E. (2022). STING agonism reprograms tumor-associated macrophages and overcomes resistance to PARP inhibition in BRCA1-deficient models of breast cancer. Nat. Commun. 13 (1), 3022. 10.1038/s41467-022-30568-1 35641483 PMC9156717

[B107] Xiong F. Q. Zhang W. Zheng C. Li Y. Gong X. Zhang Y. (2023). Gemcitabine-loaded synthetic high-density lipoprotein preferentially eradicates hepatic monocyte-derived macrophages in mouse liver with colorectal cancer metastases. Acta Pharmacol. Sin., 1–11. 10.1038/s41401-023-01110-w PMC10618456 37225846

[B108] Xu B. Sun H. Song X. Liu Q. Jin W. (2022). Mapping the tumor microenvironment in TNBC and deep exploration for M1 macrophages-associated prognostic genes. Front. Immunol. 13, 923481. 10.3389/fimmu.2022.923481 35844580 PMC9279655

[B109] Ye J. Yang Y. Dong W. Gao Y. Meng Y. Wang H. (2019). Drug-free mannosylated liposomes inhibit tumor growth by promoting the polarization of tumor-associated macrophages. Int. J. Nanomedicine 14, 3203–3220. 10.2147/IJN.S207589 31118632 PMC6509939

[B110] Ye J. Yang Y. Jin J. Ji M. Gao Y. Feng Y. (2020). Targeted delivery of chlorogenic acid by mannosylated liposomes to effectively promote the polarization of TAMs for the treatment of glioblastoma. Bioact. Mater 5 (3), 694–708. 10.1016/j.bioactmat.2020.05.001 32478203 PMC7248290

[B111] Yu C. Liu X. Yang J. Zhang M. Jin H. Ma X. (2019). Combination of immunotherapy with targeted therapy: theory and practice in metastatic melanoma. Front. Immunol. 10, 990. 10.3389/fimmu.2019.00990 31134073 PMC6513976

[B112] Yu M. Romer K. A. Nieland T. J. Xu S. Saenz-Vash V. Penman M. (2011). Exoplasmic cysteine Cys384 of the HDL receptor SR-BI is critical for its sensitivity to a small-molecule inhibitor and normal lipid transport activity. Proc. Natl. Acad. Sci. U. S. A. 108 (30), 12243–12248. 10.1073/pnas.1109078108 21746906 PMC3145699

[B113] Zamanian-Daryoush M. Lindner D. Tallant T. C. Wang Z. Buffa J. Klipfell E. (2013). The cardioprotective protein apolipoprotein A1 promotes potent anti-tumorigenic effects. J. Biol. Chem. 288 (29), 21237–21252. 10.1074/jbc.M113.468967 23720750 PMC3774392

[B114] Zamanian-Daryoush M. Lindner D. J. Buffa J. Gopalan B. Na J. Hazen S. L. (2020). Apolipoprotein A-I anti-tumor activity targets cancer cell metabolism. Oncotarget 11 (19), 1777–1796. 10.18632/oncotarget.27590 32477466 PMC7233810

[B115] Zhang J. Qu C. Li T. Cui W. Wang X. Du J. (2019). Phagocytosis mediated by scavenger receptor class BI promotes macrophage transition during skeletal muscle regeneration. J. Biol. Chem. 294 (43), 15672–15685. 10.1074/jbc.RA119.008795 31462534 PMC6816089

[B116] Zhao Y. Wang H. Yang Y. Jia W. Su T. Che Y. (2020). Mannose-modified liposome Co-delivery of human papillomavirus type 16 E7 peptide and CpG oligodeoxynucleotide adjuvant enhances antitumor activity against established large TC-1 grafted tumors in mice. Int. J. Nanomedicine 15, 9571–9586. 10.2147/IJN.S275670 33293808 PMC7718974

[B117] Zheng C. Zhang W. Wang J. Zhai Y. Xiong F. Cai Y. (2022). Lenvatinib- and vadimezan-loaded synthetic high-density lipoprotein for combinational immunochemotherapy of metastatic triple-negative breast cancer. Acta Pharm. Sin. B 12 (9), 3726–3738. 10.1016/j.apsb.2022.02.021 36176911 PMC9513558

