# Supplementary material for: Mannose-functionalization of reconstituted high-density lipoprotein nanoparticles improves payload delivery and enhances M2-to-M1 phenotype reprogramming of RAW 264.7 macrophages polarized by B16-F10 melanoma cells
Source: Front Drug Deliv. 2023 Oct 24;3:1281066. doi: 10.3389/fddev.2023.1281066 (PMC12363333; doi:10.3389/fddev.2023.1281066)
Supplement: Supplementary file 1 [file Presentation1.pdf]

Supplementary Material

Mannose-functionalization of reconstituted high-density lipoprotein nanoparticles improves payload delivery and enhances M2-to-M1 phenotype reprogramming of RAW 264.7 macrophages polarized by B16-F10 melanoma cells

Akpedje S. Dossou, Morgan E. Mantsch, Nirupama Sabnis, Rance E. Berg*, Rafal Fudala, Andras G. Lacko*

*** Correspondence:** Andras G. Lacko: andras.lacko@unthsc.edu

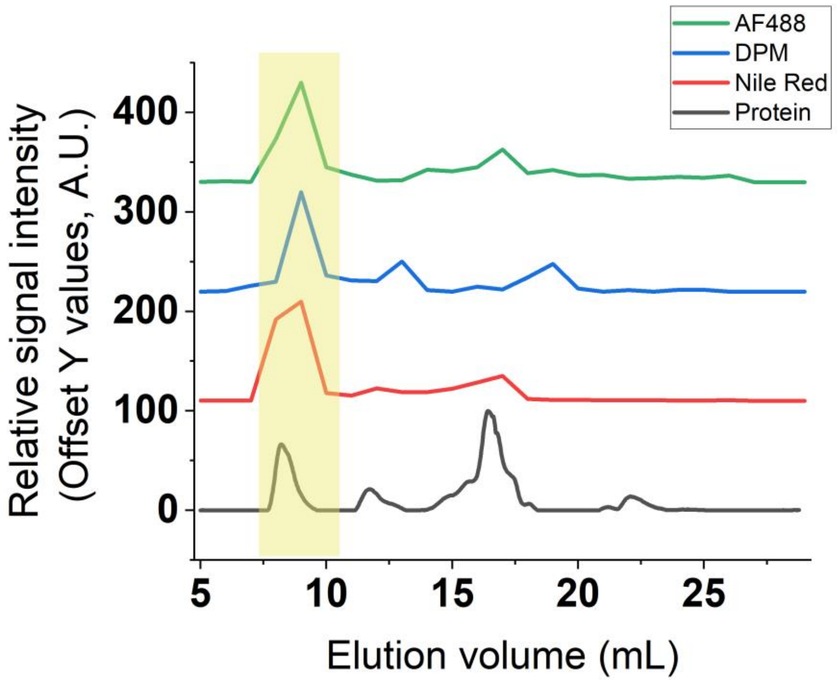

Supplementary Figure 1: Analysis of fast-protein liquid chromatography from the incubation of rHDL(ApoA-I-AF488)-DPM-NR NPs in serum. The nanoparticles were incubated in fetal bovine serum for 24 hours at 37 degrees Celsius. After incubation, the sample was loaded into an AKTA FPLC chromatography system and eluted with PBS at a flow rate of 0.5 mL/min through column (superose 6 Increase 10/300, Sigma Aldrich). The UNICORN 5 software (Cytiva, Marlborough, MA, USA) was utilized to visualize and process the elution profile. The black line represents FPLC trace of the proteins obtained via the 280 nm absorbance detector of the FPLC system. The, 100 µL of the individual eluted fractions were transferred to a 96-well plate to assess absorbance at 450 nm for (DPM as described in the Methods section), AF488 fluorescence (excitation 490 nm, emission 518 nm) and Nile red

fluorescence (excitation 550 nm, emission 620 nm). The yellow segment designates the FPLC fraction where the rHDL-DPM NPs usually elute.

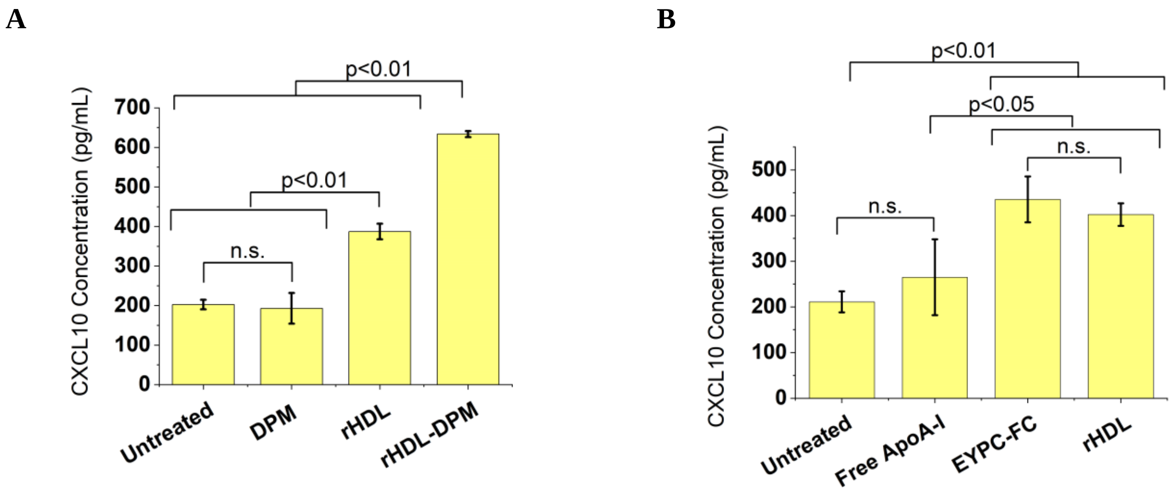

Supplementary Figure 2: Contribution of components of the rHDL-DPM NPs in the stimulation of CXCL10 production in the B16-F10 CM-educated RAW 264.7 macrophages. (**A**) CXCL10 production in the B16F10 CM-educated RAW 264.7 macrophages with treatment of DPM micelles, rHDL NPs and rHDL-DPM NPs (n.s., non-significant) (**B**) Contribution of components of the rHDL NPs in stimulating CXCL10 production in the B16-F10 CM-educated RAW 264.7 macrophages. EYPC: egg yolk phosphatidylcholine; FC: free cholesterol. All data are graphed as mean ± SD of three independent experiments.

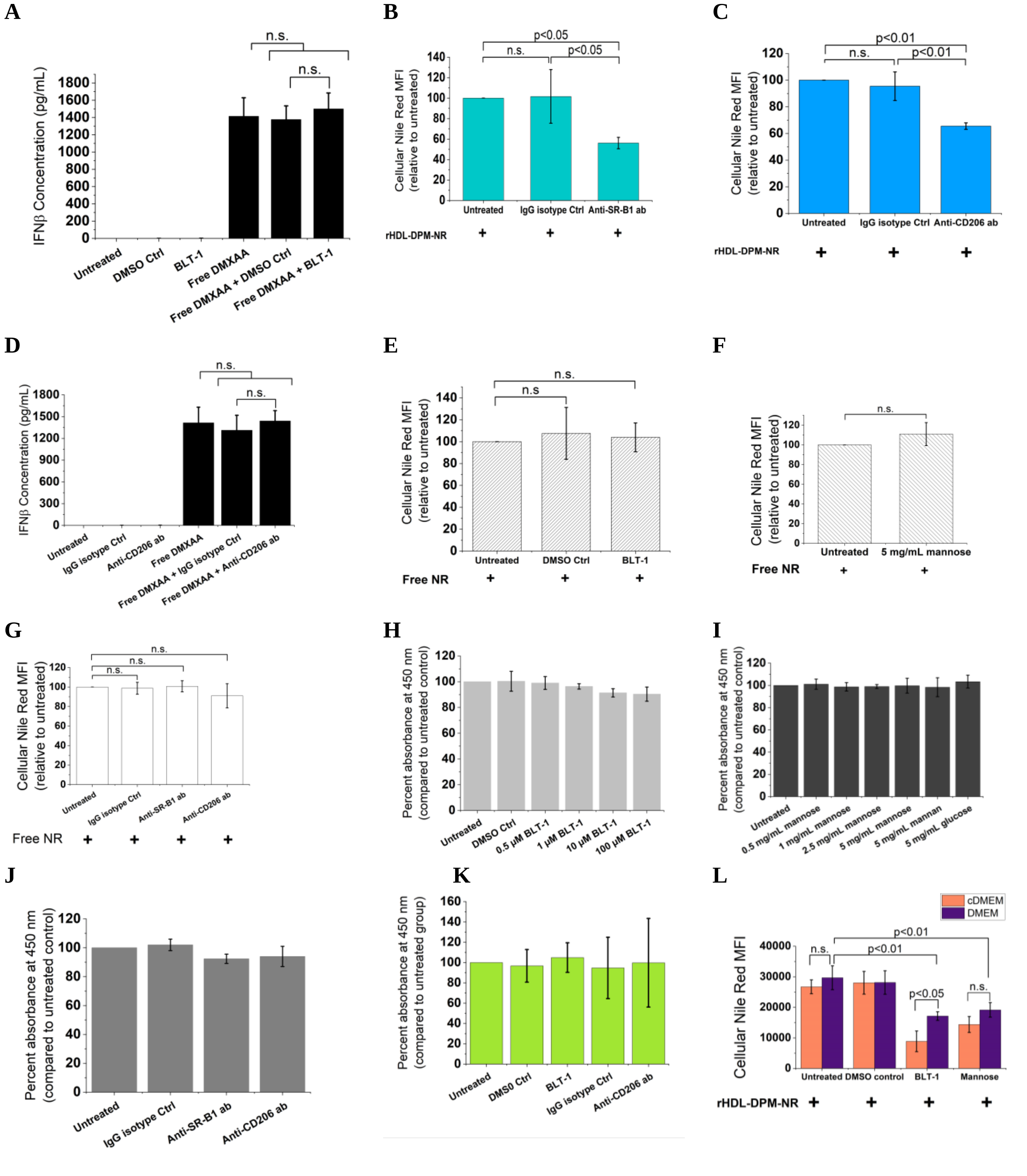

Supplementary Figure 3: The SR-B1 and CD206 inhibitors do not affect the uptake of the free payload (DMXAA or NR). **(A)** Effect of BLT-1 on DMXAA-mediated IFNβ production (assayed via ELISA) when B16-F10 CM-treated RAW 264.7 macrophages are treated with the free DMXAA (n.s., non-significant) **(B)** Effect of anti-SR-B1 blocking antibody on NR uptake from rHDL-DPM-NR NPs. **(C)** Effect of anti-CD206 blocking antibody on NR uptake from rHDL-DPM-NR NPs. **(D)** Effect of

anti-CD206 antibody on DMXAA-mediated IFNβ production (assayed via ELISA) when B16-F10 CM-treated RAW 264.7 macrophages are treated with the free DMXAA **(E-G)** Effect of the receptor inhibitors on NR uptake when the cells are incubated with Free NR instead of the rHDL-DPM-NRs. **(H-J)** Cytotoxic effect of the different inhibitors on the B16-F10 CM-educated RAW 264.7 macrophages as assessed by CCK8 assay described in the Materials and Methods section. The cells were incubated with 10 µM BLT-1 treatments and the DMSO control for 1 hour, the mannose, mannan and glucose treatments for 15 min, and the antibodies for 1 hour before addition of the CCK8 reagent. **(K)**. Evaluation of cytotoxic effects of the inhibitors after 24 hours of incubation with the B16-F10 CM-educated RAW 264.7 macrophages. For the 24-hour time point, 1 µM BLT-1 was used instead of 10 µM BLT-1 based on IC50 values obtained from the treatment of B16-F10 CM-educated RAW 264.7 macrophages with a range BLT-1 concentrations. **(L)** Effect of BLT-1 (10µM) and mannose (5 mg/mL) on NR uptake when the experiment is conducted with serum-free DMEM or cDMEM. The data is presented as mean ± SD of three independent experiments.

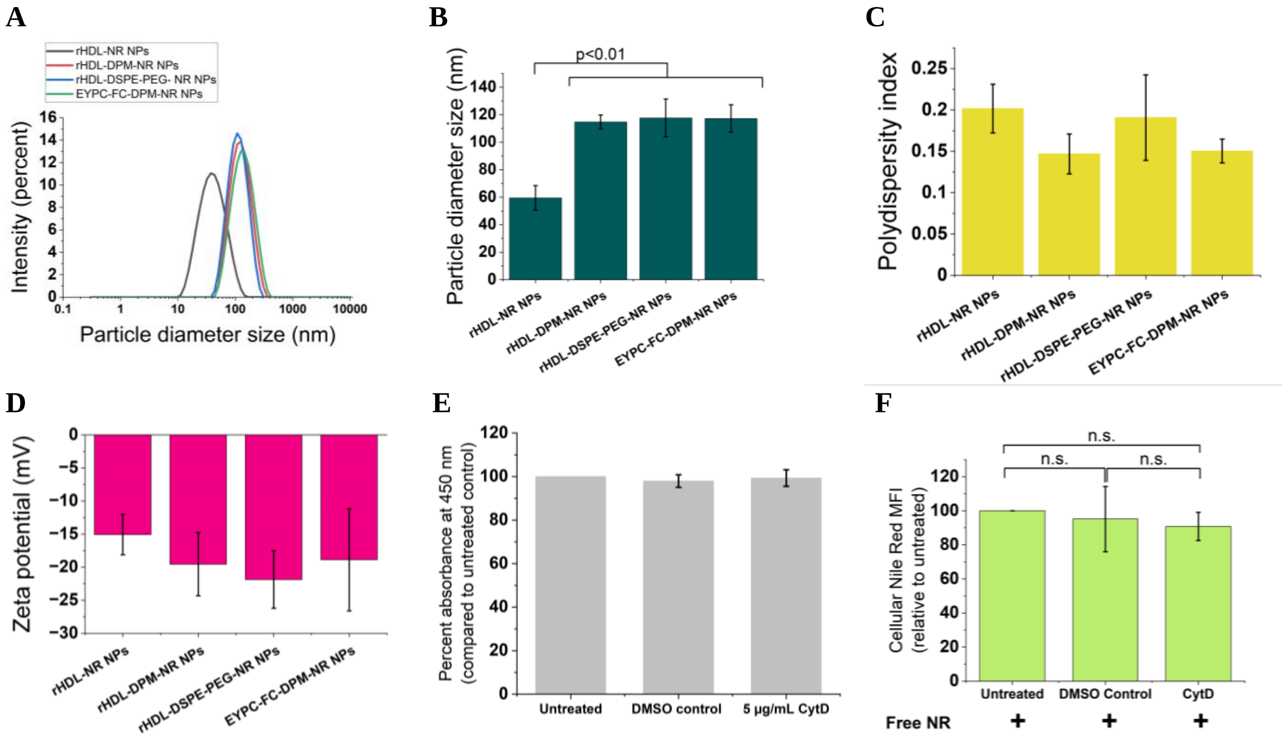

Supplementary Figure 4: Characterization of ApoA-I and mannose moiety-free particles and effect CytD on NR uptake with Free NR. **(A)** Intensity distribution of particle sizes. **(B)** Z-average of particle diameter size. **(C)** Polydispersity index to assess particle homogeneity in size. **(D)** Zeta potential of NR formulations. **(E)** Evaluation of cytotoxic effect of the 5-hour treatment of the B16-F10 CM-educated RAW 264.7 macrophages with 5 µg/mL CytD. (**F**) Effect of pre-treatment with 5 µg/mL CytD on NR uptake with the Free NR in B16-F10 CM-educated RAW 264.7 macrophages (n.s, non-significant). All data are presented as mean ± SD of three independent experiments.

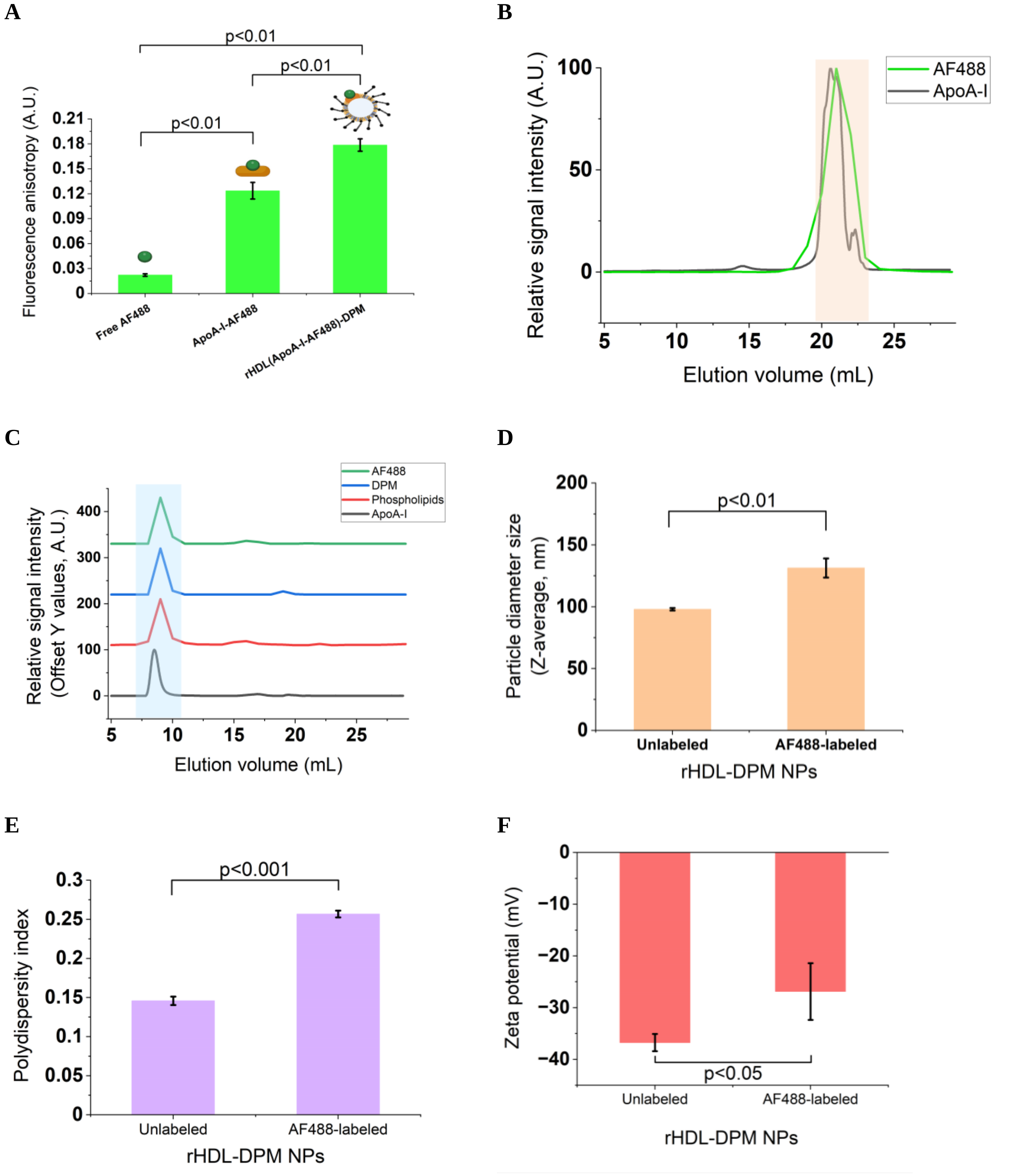

Supplementary Figure 5: Characterization of the rHDL(ApoA-I-AF488)-DPM NPs. **A)** Steady-state fluorescence anisotropy (r) of AF488, ApoA-I-AF488 and rHDL(ApoA-I-AF488)-DPM NPs, all in 1X PBS (excitation 490 nm, emission 518 nm) The Varian Cary Eclipse fluorescence spectrophotometer

(Varian Inc., Palo Alto, CA, USA) was utilized to obtain the fluorescence intensities from polarized light. Then r was calculated using the following formula: $r=(I_{VV}-GI_{VH})/(I_{VV} + 2GI_{VH})$ where I represents fluorescence intensity from vertical (v) or horizontal (H) light polarization, and G represents the ratio between I_{HV} and I_{HH}. **B)** FPLC profile of ApoA-I-AF488. **(C)** FPLC profile of rHDL(ApoA-I-AF488)-DPM NPs including detection of protein, phospholipids, DPM and AF488. The colorimetric assay Wako phospholipid C (absorbance at 590 nm, Wako Diagnostics Life Sciences, Richmond, VA, USA) was utilized to detect phospholipids in each FPLC fraction. **(D-F)** Comparison of particle diameter size (z-average), polydispersity index and zeta potential. The bar graphs are presented as mean ± SD of three independent experiments.
